# Synergistic anti-proliferative activity of JQ1 and GSK2801 in triple-negative breast cancer

**DOI:** 10.1186/s12885-022-09690-2

**Published:** 2022-06-08

**Authors:** Nanda Kumar Yellapu, Thuc Ly, Mihaela E. Sardiu, Dong Pei, Danny R. Welch, Jeffery A. Thompson, Devin C. Koestler

**Affiliations:** 1 Department of Biostatistics & Data Science, University of Kansas, Medical Center, KS Kansas City, USA; 2 The University of Kansas Cancer Center, Kansas City, KS USA; 3 Department of Cancer Biology, University of Kansas, Medical Center, KS Kansas City, USA; 4 Departments of Molecular & Integrative Physiology and Internal Medicine, University of Kansas, Medical Center, KS Kansas City, USA

**Keywords:** Breast cancer, RNASeq, Differential expression analysis, Drug resistance, Expression studies, MTT assay

## Abstract

**Background:**

Triple-negative breast cancer (TNBC) constitutes 10–20% of breast cancers and is challenging to treat due to a lack of effective targeted therapies. Previous studies in TNBC cell lines showed in vitro growth inhibition when JQ1 or GSK2801 were administered alone, and enhanced activity when co-administered. Given their respective mechanisms of actions, we hypothesized the combinatorial effect could be due to the target genes affected. Hence the target genes were characterized for their expression in the TNBC cell lines to prove the combinatorial effect of JQ1 and GSK2801.

**Methods:**

RNASeq data sets of TNBC cell lines (MDA-MB-231, HCC-1806 and SUM-159) were analyzed to identify the differentially expressed genes in single and combined treatments. The topmost downregulated genes were characterized for their downregulated expression in the TNBC cell lines treated with JQ1 and GSK2801 under different dose concentrations and combinations. The optimal lethal doses were determined by cytotoxicity assays. The inhibitory activity of the drugs was further characterized by molecular modelling studies.

**Results:**

Global expression profiling of TNBC cell lines using RNASeq revealed different expression patterns when JQ1 and GSK2801 were co-administered. Functional enrichment analyses identified several metabolic pathways (i.e., systemic lupus erythematosus, PI3K-Akt, TNF, JAK-STAT, IL-17, MAPK, Rap1 and signaling pathways) enriched with upregulated and downregulated genes when combined JQ1 and GSK2801 treatment was administered. RNASeq identified downregulation of *PTPRC, MUC19, RNA5-8S5, KCNB1, RMRP, KISS1* and *TAGLN* (validated by RT-qPCR) and upregulation of *GPR146, SCARA5, HIST2H4A, CDRT4, AQP3, MSH5-SAPCD1, SENP3-EIF4A1, CTAGE4* and *RNASEK-C17orf49* when cells received both drugs. In addition to differential gene regulation, molecular modelling predicted binding of JQ1 and GSK2801 with PTPRC, MUC19, KCNB1, TAGLN and KISS1 proteins, adding another mechanism by which JQ1 and GSK2801 could elicit changes in metabolism and proliferation.

**Conclusion:**

JQ1-GSK2801 synergistically inhibits proliferation and results in selective gene regulation. Besides suggesting that combinatorial use could be useful therapeutics for the treatment of TNBC, the findings provide a glimpse into potential mechanisms of action for this combination therapy approach.

**Supplementary Information:**

The online version contains supplementary material available at 10.1186/s12885-022-09690-2.

## Introduction

Breast cancer is the most common cancer in women worldwide, aside from skin cancer, accounting for over 1.4 million cases annually [1–4]. From 1975 to 2010, the mortality rate of breast cancer declined by 34%, from 32 per 100,000 per year in 1975 to 21 per 100,000 per year in 2010. However, the incidence of localized breast cancers increased by 30% in the same time frame with no commensurate decline in the number of regional breast cancers [5]. After 2010, mortality rates continued to decline by 1.2%-2.2% per year in women aged 40–79 years, but increased by 2.8% per year in women aged 20–29 years and 0.3% per year in women aged 30–39 years [6, 7]. Existing therapies for breast cancer exhibit several different mechanisms of action, including damaging DNA (Cisplatin, Etoposide and Bleomycin), targeting overexpression of receptors (Tamoxifen, Trastuzimab, Cetuximab, Gefitinib and Imatinib) and inhibiting intracellular signal transduction (Rapamycin). While such therapeutic and intervention procedures have reduced mortality, overall survival rates suggest that more aggressive treatments and/or interventions may be needed [8].

Among breast cancers, Triple-Negative Breast Cancer (TNBC) represents a high-risk breast cancer because of its poor response to specific therapies [9]. TNBC accounts for a non-negligible proportion of breast cancers, accounting for 10–20% of all breast cancers [10]. TNBC is typically managed with standard, non-targeted treatment procedures [11, 12]; however, such treatments tend to be associated with high rates of relapse [10, 11]. The reason for the lack of success of current treatments is due to a poor understanding of the molecular mechanisms underlying TNBC. TNBC is devoid of the three major receptors: estrogen receptor (ER), progesterone receptor (PR), and human epidermal growth factor receptor 2 (HER2) [13]. These three receptors are the main targets for several breast cancer therapeutics [14] and absence of the aforementioned receptors leads to development of drug resistance [15, 16]. Furthermore, TNBC is often considered more aggressive, more likely to recur, has a worse prognosis, and disproportionally affects younger women compared to other breast cancer subtypes [17]. The standard of care for TNBC is cytotoxic chemotherapy; unfortunately, the development of drug resistance is common [15, 16]. The complex molecular heterogeneity of TNBC [18] means that a range of targeted therapies will likely be needed to continue to make progress in treating this disease. Therefore, it is critical to continue the search for novel therapeutic strategies and identify their targets so that appropriate biomarkers can be used to make personalized treatment decisions and ultimately, improve the prognosis associated with TNBC.

While still in the early stages of investigation, small molecule bromodomain inhibitors (BDI) that reduce the pathogenicity of TNBC are a promising approach for treating TNBC. Bromodomains (BD) are 110 amino acid protein domains that recognize acetylated lysine residues on the N-terminal tails of histones [19, 20]. BD represent “readers of lysine acetylation” and are responsible for signal transduction via acetylated lysines. Since lysine acetylation is prerequisite for histone association and chromatin remodeling, BDI represent a promising and emerging agent for treating TNBC. In recent years there is an increasing popularity of BDI for their effective anti-cancer activity and have emerged as a promising class of anti-cancer drugs. CDK4/6 inhibitors and paclitaxel have higher synergies with bromodomain-extra-terminal domain (BET) inhibitors against TNBC [21]. A novel ATAD2 BDI, AM879, which was discovered by Dahong et al*.*, presents potential inhibitory activity in breast cancer cells [22]. Guan-Jun et al*.* discovered a BDI which showed potential anticancer activity in NF-kappa B-active MDA-MB-231 TNBC cells [23]. Minjin et al*.* discovered five potential BRD4 based BDI with high binding affinity and their co-crystal structures experimentally demonstrated impressive inhibitory activity and mode of action for the treatment of cancers [24]. TRIM24 BD is a therapeutic target for several cancers including breast cancer for which Qingqing et al*.* developed potential BDI based on N-benzyl-3,6-dimethylbenzo[d]isoxazol-5-amine derivatives [25]. CREB (cyclic-AMP response element binding protein) binding protein (CBP) bromodomain is related to several human malignancies for which a potential BDI, DC-CPin734 was discovered by Xiaoyang et al*.* [26]. Such findings suggest that BDI have distinct clinical efficacies in cancer treatments and further suggest that combinations of BDI may also exert unique effects.

Among BD-containing proteins, members of the BET domain [27], Bromo adjacent to zinc finger 2A (BAZ2A) [28] and Bromo adjacent to zinc finger 2B (BAZ2B) [29] domains play important roles in transcriptional regulation. These BD family members are potential targets in several cancer types [30]. JQ1, a BET domain inhibitor [31], and GSK2801, BAZ2A/B domain inhibitor [32], were recently tested on MDA-MB-231, HCC-1806, and SUM-159 TNBC cell line models [32]. Preclinical models were sensitive to BET-dependent TNBC inhibition [33]; however, clinical trials testing BD-based inhibition by means of BDI such as JQ1 plus GSK2801 are ongoing.

The functional distinctions in the mechanisms by which BET and BAZ2A/B domains regulate transcription machinery remain poorly defined. In order to gain insight into this question, Bevill et al*.* (2019) performed dose-dependent drug synergy screen targeting several BD families [32]. They found little or no growth inhibition as single agents. In contrast, combined treatments resulted in strong growth inhibition of TNBC. While promising, expression of specific gene elements identified via whole transcriptome profiling under different treatment conditions were not validated nor did they examine specific target candidates. While interesting, their study was limited by the use of a single concentration which did not assess cytotoxic effects on each cell line. Since cells undergoing apoptosis or other mechanisms of cell death have inherently different expression profiles, interpretation of drug-induced expression changes is limited.

To address these gaps and limitations, we expanded the work of Bevill et al*.* (2019). Specifically, we sought to shed light on the specific metabolic networks involved in TNBC inhibition, both in single and combined agent treatments which would be the novel strategy of the current work. We further wanted to explore the synergistic action of JQ1 and GSK2801 at different dose concentrations beyond the studies by Bevill et al. to optimize the lethal dose concentrations both as single and combined agents against multiple cancer cell types. Investigating the expression of specific genes associated with the synergic effect would provide more probable molecular mechanism associated with synergistic action and the enhanced cytotoxic effect. Further, adding molecular modelling studies will help to elucidate the intermolecular interactions of the drugs and provide strong evidence of their inhibitory actions.

We adopted Next-Generation Sequencing technology (NGS)-based bioinformatics approaches to identify differentially expressed genes (DEGs) across different treatment conditions using publicly available RNASeq data collected on TNBC cell lines treated with JQ1 + GSK2801, and subsequently validated differentially expressed genes through RT-qPCR. Our analyses reveal common upregulated and downregulated genes across all treatments that could be treated as therapeutic targets. Molecular modelling studies were employed to predict the drug binding interactions with the identified target proteins. Our investigations shed light on the synergy between JQ1 and GSK2801 in TNBC cell lines. Furthermore, our studies support the distinct functional mechanism of JQ1 and GSK2801 through BD inhibition, along with revealing unique adaptive mechanisms of BDI that are amenable to co-inhibition of BDs to induce TNBC inhibition across the three cell line models.

## Materials and methods

**Fig. 1:**
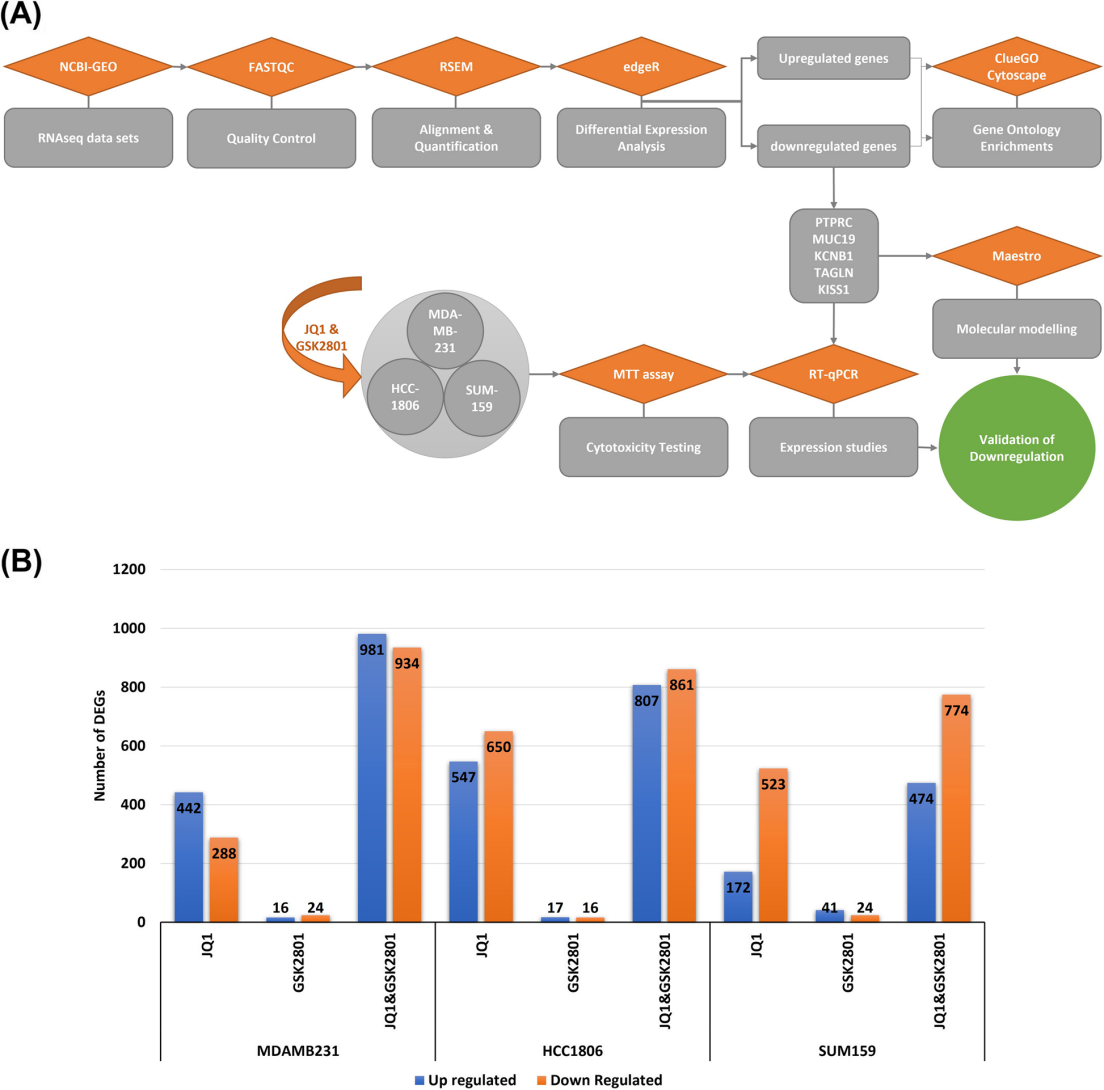
**A**. Schematic diagram of methods and tools. The steps/tools used to identify differentially expressed genes, along with their subsequent validation through computational and in vitro methods. **B**. Differential expression analysis of RNASeq data. Bar plots depicting the number of upregulated/downregulated DEGs in treated samples (JQ1, GSK2801 and JQ1&GSK2801) compared to control across different TNBC cell lines

A general overview of the analytical strategy (e.g., tools and methods) is given in Fig. 1-A. Further description of the bioinformatics and statistical methods used in our analyses are explained as follows.

### Data sources

RNASeq expression data collected on MDA-MB-231, HCC-1806, and SUM-159 TNBC cell lines treated with JQ1 and GSK2801 alone and in combination for 72 h [34] were retrieved from Gene Expression Omnibus (GEO) database (GEO series ID: GSE116907). RNASeq data were prepared on Illumina NextSeq 500 using high-throughput sequencing technology and included 12 short read archive (SRA) experimental datasets. Characteristics of the experimental design are represented in supplementary material (S1 File-Table S1**)**.

### Data processing and generation of gene counts

The quality of the sequencing reads was assessed using FastQC (https://www.bioinformatics.babraham.ac.uk/projects/fastqc/). Low quality reads with poor base sequence quality and adaptors were removed using the Trimmomatic tool [35]. After removing poor quality reads and trimming adaptors, the remaining reads were subjected to further analysis. All the processed datasets were quantified using the RSEM tool [36]. The human hg38 reference genome was used and the genome indices were constructed using Bowtie2 [37]. The gene count result files generated from RSEM analysis were used for downstream differential expression analyses.

### Identification of differentially expressed genes (DEGs)

We aimed to derive the drug induced response in each TNBC cell line model and hence, treatment wise differential expression analysis was carried out. For each cell line there was one control and three treated samples (JQ1, GSK2801 and JQ1 + GSK2801) making a total of 12 biological replicates across three TNBC cell lines. Differentially expressed genes were identified comparing control vs JQ1, control vs GSK2801 and control vs JQ1 + GSK2801. The quantified gene counts matrix was used to derive the differential expression patterns in the R-environment using the edgeR package [38] in Bioconductor. Genes with zero counts in all the samples were excluded prior to analysis. The data were normalized using the trimmed mean of M-values (TMM) method both within and between samples. Inter-sample relationships were examined by producing a plot based on multidimensional scaling. The primary parameters such as log2 fold change (logFC), log counts per million (logCPM), and *p*-value were derived for each gene and for each of the four comparisons of interest. Genes with log2 fold change > 1 or < -1 and *p*-values < 0.05 were considered as significantly differentially expressed genes (DEGs) and formed the basis for downstream analyses.

### Gene functional enrichment analysis

Gene ontology functional annotations and pathways enriched with statistically significant DEGs were analyzed by ClueGO [39] using the Cytoscape software environment [40]. ClueGO V.2.5.7 plugin was installed on Cytoscape V.3.7.2 and the genes were queried against updated versions of Kyoto Encyclopedia of Genes and Genomes (KEGG) [41]. The biological terms were derived for the large clusters of DEGs using functional enrichment analysis and grouped into networks/metabolic pathways for upregulated and downregulated genes separately. A two-sided (Enrichment/Depletion) test based on hypergeometric distribution was used for the gene ontology analysis. The Bonferroni step down method was used for correction with the threshold *p*-value of 0.05.

### Molecular modelling studies

#### Homology modelling and protein processing

The protein sequences of the five downregulated proteins PTPRC (NP_002829.3), MUC19 (NP_775871.2), KCNB1 (XP_006723847.1), TAGLN (NP_001001522.1) and KISS1 (NP_002247.3) were retrieved from National Center for Biotechnology Information (NCBI). Three dimensional models were constructed through automated homology modelling using Modweb server where the structures were built based on the template homology (https://modbase.compbio.ucsf.edu/modweb/). The stereo chemical quality of the homology models was validated by Ramachandran plots [42]. The validated models were further processed individually using the pre-process module of Schrödinger Maestro 12.6 version [43]. Bond orders were assigned in the protein and hydrogens were added. Disulfide linkages were created at the possible locations. Missing side chains and missing loops were added. Water molecules which are beyond 5 Å were removed. All the proteins were prepared by restrained minimization using optimized potentials for liquid simulations-3e (OPLS3e) force field [44].

#### Ligand preparation

The three-dimensional co-ordinates of JQ1 (49,871,818) and GSK2801 (73,010,930) were retrieved from the NCBI-PubChem database and optimized in the Maestro graphical environment. The structures were processed independently using the Ligprep module under OPLS3e force field. The ligands were subjected to ionization and possible states were generated at pH 7 ± 2 using Epik. Desalting was done and the tautomers were generated. The ligands were investigated for their hydrogen bonding efficiency, hydrophobic surface area and electron density clouds around the molecules, to predict their efficiency of interaction with the target proteins at the binding sites.

#### Molecular docking

Individual docking reactions were carried out for each protein with JQ1 and GSK2801 ligands. The optimized conformations of the proteins were loaded into Maestro workspace and the protein receptor grids were generated using the glide tool. Grid centers were determined from the active residues of the receptor proteins. Glide’s ligand docking module was used to dock JQ1 and GSK2801 into the specified binding grids of the PTPRC, MUC19, KCNB1, TAGLN, and KISS1 proteins [45]. The standard precision (SP) docking mode was used for the flexible ligand sampling without smearing any constraint. The binding efficiency and ligand affinities were assessed as docking scores and the docked ligand poses were analyzed and visualized by Maestro interface.

The docking score was calculated as follows:

$$Docking\,score\, = \,a\, \times \,vdW\, \times \,Coul\, + \,Hbond\, + \,Lip\, + \,BuryP\, + \,RotB\, + \,Site$$

a & b = co-efficient constant for vdW and Coul respectively.

vdW = van der Waals energy

Coul = Coulomb energy

Hbond = Hydrogen bonding with receptor

Metal = Binding with metal

Lipo = Constant term for lipophilic

BuryP = Buried polar group penalty

RotB = Rotatable bond penalty

Site = active site polar interaction

### In vitro* studies*

#### Cells and cell cultures

MDA-MB-231, HCC-1806 and SUM-159 are widely used TNBC cell line models [46]. MDA-MB-231 cells were maintained in a 1:1 mixture of Dulbecco’s MEM and Ham’s F12 media containing 5% FBS and 1X-non-essential amino acids in the absence of antibiotics. HCC-1806 cells were maintained in RPMI-1640 media with 10%-FBS and PenStrep. SUM-159 cells were maintained in Ham’s F12 media with 5% FBS, Insulin (5 ug/ml), Hydrocortisone (1 ug/ml), Gentamycin (5 ug/ml), fungizone and PenStrep. The cultures were maintained at 37 °C in a humidified CO_2_-controlled (5%) incubator. Prior to performing in vitro investigations, all cell cultures were tested using PCR assay for *Mycoplasma spp* and found to be uninfected.

#### Determination of cytotoxic effects of JQ1 and GSK2801 by MTT assay

Cells were seeded in 96 well plates (MDA-MB-231 cells, 5000 cells/well; HCC-1806 and SUM-159 cells, 8000 cells/well) in triplicate with 200 µl of media per well and allowed to adhere for 24 h [47]. JQ1 (SML1524) and GSK2801 (SML0768) were purchased from Sigma- Aldrich and dissolved in DMSO to make stock solutions (JQ1-20 mg/mL; GSK2801-10 mg/mL) and further dilutions were made with culture media immediately prior to each experiment. JQ1 and GSK2801 stock solutions were stored at 4 oC and -20 oC, respectively for no longer than 30 days. The spent medium was aspirated and the fresh medium containing drug concentrations ranging from 0.125 µM – 20 µM were used to treat the cells along with DMSO control. The combined treatments were carried out with JQ1 (25 nM, 50 nM, 125 nM, 250 nM, 500 nM, 1000 nM) combined with 10 µM and 20 µM of GSK2801 to assess the possible synergistic effects. After 72 h, spent media was aspirated; MTT was added and incubated for 3 h at 37 °C. MTT was removed, DMSO was added, and the plate was kept on shaker for 30 min at 60 RPM to dissolve crystals completely. The plate was read at 540 nm and OD recorded. Percent viability was calculated $(OD\,treated\, \div \,OD\,Control)\, \times \,100$ and the viabilities were plotted [48]. The combination index (CI) for each treatment combination was determined by the Chou-Talalay method [49] using CompuSyn software [50].

#### RT-qPCR Expression studies

RT-qPCR was used to validate the expression of *PTPRC, MUC19, KCNB1, TAGLN* and *KISS1* genes following treatment. The treatment strategic plan to evaluate the expression of these genes is summarized in Table 1. The TNBC cells were seeded in six well plates (MDA-MB-231, 150,000 cells/well; HCC-1806 or SUM-159, 240,000 cells/well) and allowed to adhere for 24 h. Spent media was aspirated and fresh media containing drugs was added and incubated for 72 h.

**Table 1:** Experimental setup for the gene expression studies

Sample No	Treatment (JQ1 / GSK2801)
1	DMSO Ctrl
2	50 nM / 0 µM
3	125 nM / 0 µM
4	250 nM / 0 µM
5	0 nM / 10 µM
6	50 nM / 10 µM
7	125 nM / 10 µM
8	250 nM / 10 µM

Samples and treatment conditions of JQ1 and GSK2801 drugs used for the evaluation of gene expression in the three TNBC cell lines.

The total RNA was isolated using Quick-RNA Miniprep Kit (Zymo research, USA-R1054) according to manufacturer instructions. Purity and concentrations of the isolated RNA were determined by NanoDrop 2000 spectrophotometer (Thermo Scientific, USA). cDNA was synthesized by reverse transcription using iScript cDNA Synthesis Kit (Bio-Rad #1,708,891). cDNA synthesis was performed with 1 µg of total RNA, 5 µl of 5X iScript reaction mix and 1 µl of iScript reverse transcriptase in a final volume of 20 µl.

Quantitative RT-PCR was performed using the SYBR™ select master mix (Applied Biosystems-4472908). The reaction was set up with 5 µl of SYBR master mix 2X, 300 nM of each primer and 100 ng of cDNA template in a total reaction volume of 10 µl. Primers were designed using Primer3 [51] online software and are shown in supplementary material (S1 File-Table S2). 18S rRNA gene was used as a standard reference to normalize the expression levels. Gene expression levels were calculated by the comparative ΔΔCt. The Ct values were obtained for the standard reference gene (18S rRNA) and the five target genes (*PTPRC, MUC19, KCNB1, TAGLN* and *KISS1*). Delta Ct (ΔCt) values of the samples were calculated by subtracting the reference gene Ct values from the Ct value of the samples. ΔCt values of the DMSO control sample were averaged to obtain an average ΔCt value. The ΔCt values of all the samples including DMSO control were then subtracted from the average ΔCt value of the DMSO control to obtain the ΔΔCt value. The relative gene expression fold was calculated by 2^−ΔΔCt^ formula for DMSO control and treated samples. The values of each sample group were then averaged and presented as relative fold gene expression [52].

#### Statistical analysis

All the results are displayed as mean ± SD, calculated from three independent experiments. Statistical significance between experimental conditions was determined using two-tailed, two-sample Student's t-tests and a non-parametric Wilcoxon rank sum test. A significance level of *p*-value < 0.05 was considered statistically significant.

## Results

### Differential expression analysis using publicly available RNASeq data sets

To explore the molecular mechanisms by which JQ1 and GSK2801 exert anti-proliferative activity in TNBC cell lines, we first performed a differential expression analysis using the RNASeq data obtained from the Bevill et al. (2019) study [32]. Expression profiles were determined via RNASeq analysis of MDA-MB-231, HCC-1806 and SUM-159 cell lines treated with JQ1 and GSK2801 individually, and in combination. The gene count matrix generated from the RSEM analysis of the RNASeq data is provided as supplementary material (S2 File). After normalization, data were visualized using boxplots (S1 File-Fig. S1-A & B), heatmaps (S1 File-Fig. S1-C & D) and Multidimensional Scaling (MDS) plots (S1 File-Fig. S2) to understand the sources of variation across samples. Common and tagwise dispersions were estimated, exact tests were performed and differences in gene expression between treated and control were determined in each cell line model and across all treatment conditions. The overall differential expression in each cell line model was determined in terms of number of DEGs and summarized in Fig. 1-B.

A list of DEGs for each treatment/treatment combination and within each TNBC cell line are provided as supplementary material S3 File. The corresponding smear and volcano plots representing the DEGs are also provided in the supplementary material (S1 File-Tables S3-S5).

*MDA-MB-231:* A total of 730 DEGs were observed in cells treated with JQ1, among which 442 were upregulated and 288 were downregulated. In contrast, 40 DEGs in cells treated with GSK2801, among which 16 were upregulated and 24 were downregulated. A total of 1915 DEGs were observed in cells treated with both JQ1 and GSK2801 (combined treatment), of which 981 were upregulated 934 were downregulated.

*HCC-1806:* There were 1197 DEGs were observed in cells treated with JQ1, of which 547 were upregulated and 650 downregulated. GSK2801 treated cells showed 33 DEGs, among which 17 upregulated and 16 were downregulated in treated versus control cells. Treatment with both JQ1 and GSK2801 resulted in 1668 DEGs, among which 807 were upregulated 861 were downregulated.

*SUM-159:* A total of 695 DEGs were identified when comparing JQ1 treated and control SUM-159 cells, among which 172 were upregulated and 523 were downregulated in JQ1 treated cells. GSK2801 treated SUM-159 cells showed 65 DEGs, among which 41 upregulated and 24 were downregulated in treated versus control cells. Treatment with both JQ1 and GSK2801 resulted in 1248 DEGs, among which 474 were upregulated 774 were downregulated.

Across all three TNBC cell line models, we observed a greater number of DEGs in the combined treatment as compared to the single agent treatments. To identify the key elements responsible for the anti-cancer activity of JQ1 and GSK2801, the DEGs were further analyzed by constructing network maps. DEGs that were commonly and uniquely upregulated and downregulated by the three treatment conditions were visualized using these network maps (supplementary material S4 File). MDA-MB-231 cell line models showed upregulation of *MSH5-SAPCD1* and *SENP3-EIF4A1* genes and downregulation of *RMRP*, *KISS1* and *TAGLN* across all three treatment conditions. HCC-1806 cell lines showed *CTAGE4* and *RNASEK-C17orf49* genes as commonly upregulated, however zero genes were observed to be commonly downregulated across all the three treatment conditions. SUM-159 cell lines showed upregulation of *GPR146*, *SCARA5*, *HIST2H4A*, *CDRT4* and *AQP3* and downregulation of *PTPRC*, *MUC19*, *RNA5-8S5* and *KCNB1* across all three treatment conditions. Prioritizing genes that were consistently downregulated, our in vitro investigations focused on *PTPRC, MUC19, KCNB1, TAGLN* and *KISS1* and the proteins that they encode (*RNA5-8S5* and *RMRP* are not protein coding genes*)*.

### Gene functional enrichment analysis

The functional association of the identified DEGs following different treatments was determined by enrichment analysis and the associated metabolic pathways were determined. The analysis of DEGs revealed several interesting findings regarding the potential synergistic action of JQ1 and GSK2801 across the three different cancer cell lines.

**Fig. 2:**
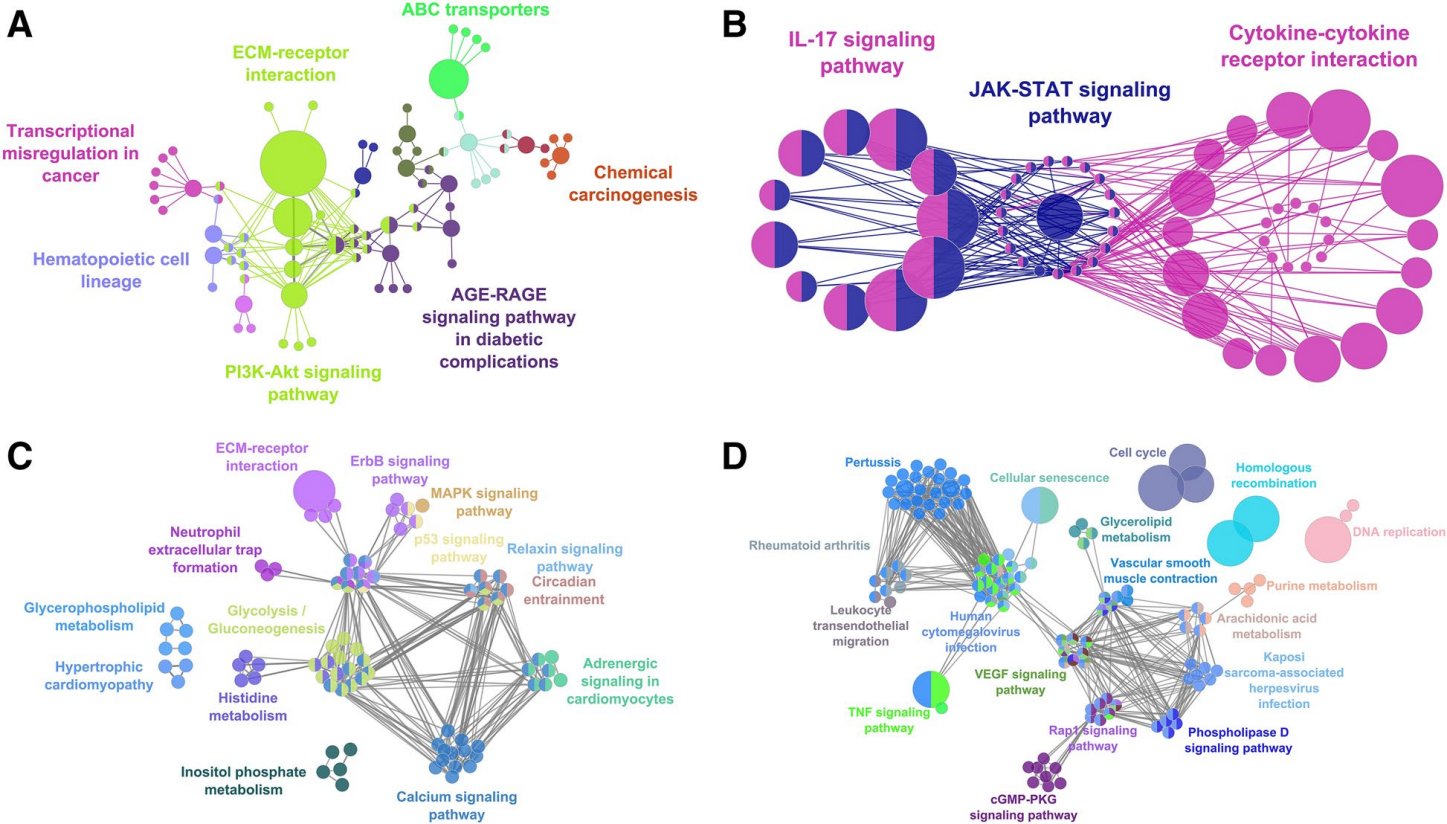
Functional enrichment analysis of DEGs identified in MDA-MB-231 cells. Metabolic pathways enriched with (**A**) upregulated (**B**) downregulated genes in JQ1 treatment. Metabolic pathways enriched with (**C**) upregulated (**D**) downregulated genes from JQ1 + GSK2801 combined treatment

*MDA-MB-231*: A total of 22 metabolic pathways were observed to be significantly enriched with genes found to be upregulated based on treatment with JQ1 (e.g., PI3K-Akt signaling pathway, Transcriptional misregulation in cancer and ECM-receptor interaction). In contrast, there were no metabolic pathways that were found to be significantly enriched with genes found to be upregulated based on treatment with GSK2801. For the combined treatment, we identified 156 pathways that were significantly enriched with upregulated genes (e.g., MAPK, Ras, Rap1 and calcium signaling pathways), and included among those 156 pathways were the 22 pathways identified from treatment with JQ1 alone. Similarly, 32 pathways (e.g., cytokine-cytokine receptor interactions, TNF, JAK-STAT and IL-17 signaling pathways) were significantly enriched with downregulated genes based on treatment with JQ1 alone, while there were no pathways found to be significantly enriched with downregulated genes based on treatment with GSK2801 alone. Finally, for the combined treatment we identified 138 metabolic pathways (e.g., cellular senescence, microRNA in cancer, NOD-like receptor signaling and AGE-RAGE signaling pathways) that were significantly enriched with the downregulated genes based on the combined treatment and which contained all 32 pathways identified when JQ1 was given alone (Fig. 2).

**Fig. 3:**
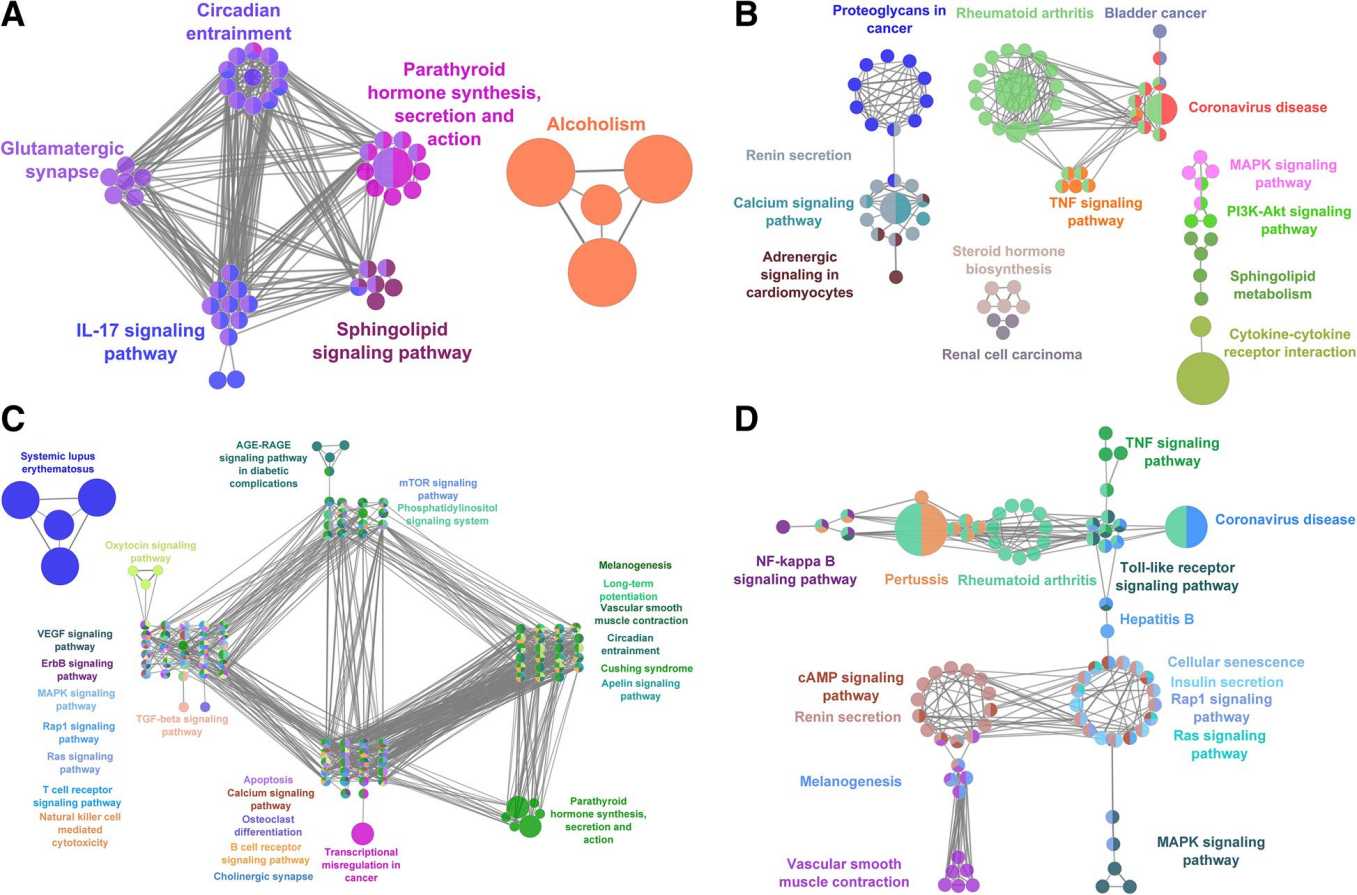
Functional enrichment analysis of DEGs identified in HCC-1806 cells. Metabolic pathways enriched with (**A**) upregulated (**B**) downregulated genes from JQ1 treatment. Metabolic pathways enriched with (**C**) upregulated (**D**) downregulated genes from JQ1 + GSK2801 combined treatment

*HCC-1806*: A total of 68 pathways were significantly enriched with genes upregulated following treatment with JQ1 (e.g., IL-17, sphingolipid signaling and circadian entrainment pathways); GSK2801 none. For the combined treatment, we identified 150 pathways that were significantly enriched with upregulated genes (e.g., AGE-RAGE, mTOR, B-cell receptor, VEGF signaling pathways), and included among those 150 pathways were the 66 pathways identified from treatment with JQ1 alone. Similarly, 108 pathways (e.g., TNF, MAPK, PI3-Akt and calcium signaling pathways) were significantly enriched with downregulated genes based on treatment with JQ1 alone, while there were no pathways significantly enriched with downregulated genes based on treatment with GSK2801 alone. Finally, for the combined treatment we identified 133 pathways (e.g., NF-kappa B, cAMP, Rap1 and Ras signaling pathways) that were significantly enriched with the downregulated genes based on the combined treatment and which contained 98 of the 108 pathways identified when JQ1 was given alone (Fig. 3).

**Fig. 4:**
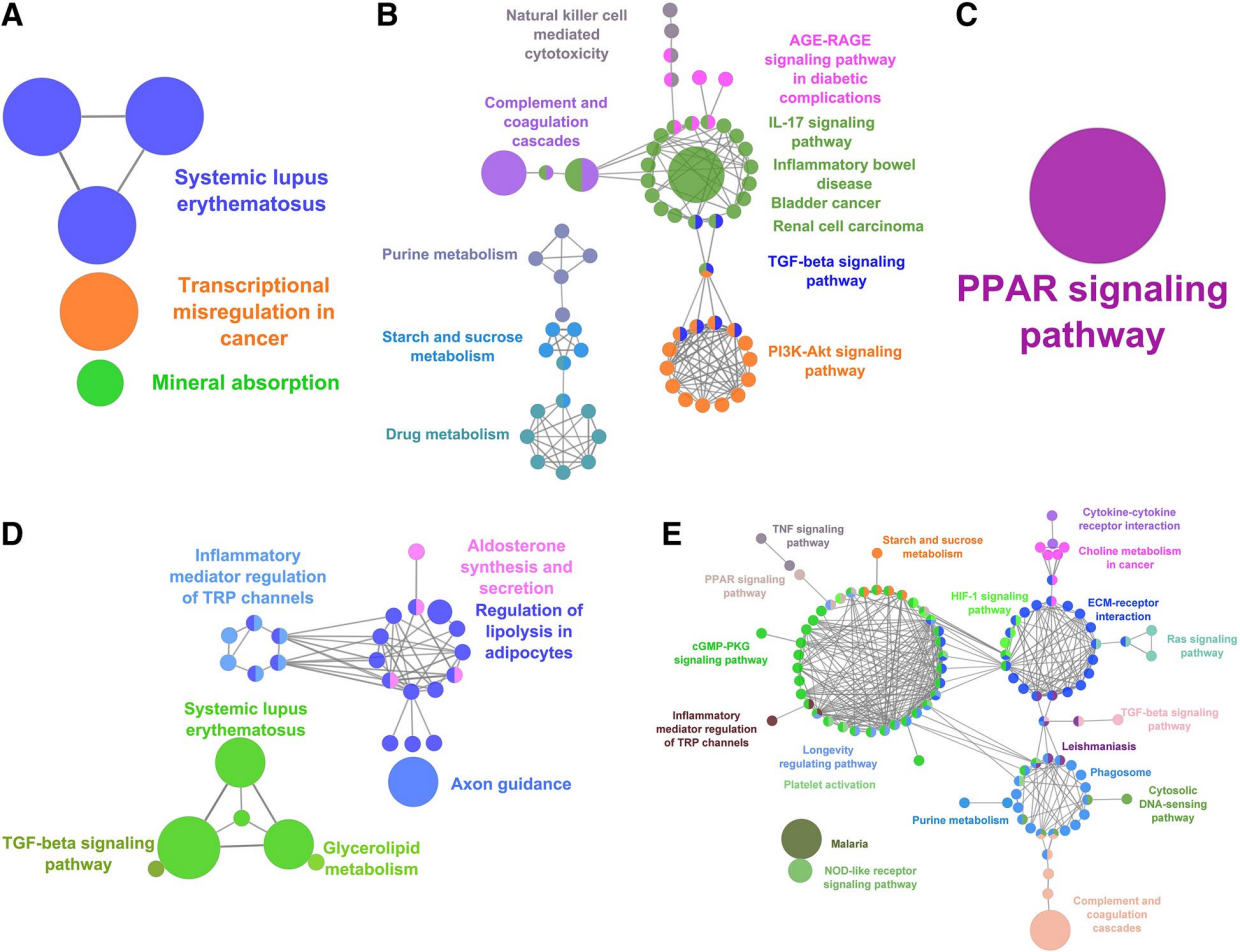
Functional enrichment analysis of DEGs from identified from SUM-159 cells. Metabolic pathways enriched with (**A**) upregulated (**B**) downregulated genes from JQ1 treatment. Metabolic pathways enriched with (**C**) upregulated genes from GSK2801 treatment. (**D**) upregulated and (**E**) downregulated genes from JQ1 + GSK2801 combined treatment

*SUM-159*: A total of 5 pathways were significantly enriched with genes found to be upregulated based on treatment with JQ1 in SUM-159 cells (e.g., systemic lupus erythematosus, Transcription misregulation in cancer and mineral absorption). For GSK2801, we identified a single pathway (PPAR signaling pathway) that was significantly enriched with upregulated DEGs when GSK2801 was administered alone. For the combined treatment, we identified 41 pathways that were significantly enriched with upregulated genes (e.g., TGF-beta signaling, aldosterone synthesis and regulation of lipolysis in adipocytes), which included the five pathways identified when JQ1 was administered alone, along with the PPAR signaling pathway that was identified when GSK2801 was administered alone. A total of 91 pathways (e.g., AGE-RAGE, IL-7, TGF-beta signaling pathways) were significantly enriched with downregulated genes based on treatment with JQ1 alone, while there were no pathways significantly enriched with downregulated genes based on treatment with GSK2801 alone. Finally, for the combined treatment we identified 131 pathways (e.g., ECM-receptor interaction, PPAR, TNF, cGMP-PKG signaling pathways) that were significantly enriched with the downregulated genes based on the combined treatment and which contained 85 of the 91 pathways identified when JQ1 was given alone (Fig. 4).

The nodes representing more than four genes are shown in each metabolic network map and the complete list of metabolic pathways enriched with upregulated and downregulated genes identified for each treatment condition and cell line are provided as supplementary material S5 File. To identify shared and unique numbers of pathways across different treatments and cell lines, pathways were represented as Venn diagrams (Fig. S3). Based on combined treatment, we found 31 pathways enriched with upregulated genes and 66 pathways enriched with downregulated genes that were common across all three cell line models (Table 2).

**Table 2:** Metabolic pathway enrichment

Upregulated	Downregulated	
Transcriptional misregulation in cancer	Cytokine-cytokine receptor interaction	Tuberculosis
ABC transporters	NOD-like receptor signaling pathway	Axon guidance
Alcoholism	Lipid and atherosclerosis	Calcium signaling pathway
Axon guidance	Amoebiasis	Phospholipase D signaling pathway
Viral carcinogenesis	TNF signaling pathway	Cholinergic synapse
Cell adhesion molecules	Influenza A	Wnt signaling pathway
Rap1 signaling pathway	JAK-STAT signaling pathway	cGMP-PKG signaling pathway
Neutrophil extracellular trap formation	Rheumatoid arthritis	Leishmaniasis
Fluid shear stress and atherosclerosis	Hematopoietic cell lineage	Complement and coagulation cascades
Necroptosis	IL-17 signaling pathway	Toxoplasmosis
Systemic lupus erythematosus	AGE-RAGE signaling pathway in diabetic complications	ECM-receptor interaction
Glycerolipid metabolism	Pertussis	Morphine addiction
Platelet activation	Inflammatory bowel disease	Inflammatory mediator regulation of TRP channels
Parathyroid hormone synthesis, secretion and action	C-type lectin receptor signaling pathway	Melanogenesis
Ferroptosis	Legionellosis	HIF-1 signaling pathway
Aldosterone synthesis and secretion	NF-kappa B signaling pathway	Relaxin signaling pathway
PPAR signaling pathway	Toll-like receptor signaling pathway	Natural killer cell mediated cytotoxicity
TGF-beta signaling pathway	Chagas disease	Vascular smooth muscle contraction
Osteoclast differentiation	Gastric acid secretion	Fluid shear stress and atherosclerosis
Estrogen signaling pathway	Viral protein interaction with cytokine and cytokine receptor	RIG-I-like receptor signaling pathway
Inflammatory mediator regulation of TRP channels	Cytosolic DNA-sensing pathway	Leukocyte transendothelial migration
Steroid hormone biosynthesis	African trypanosomiasis	Bladder cancer
Circadian entrainment	Malaria	Arachidonic acid metabolism
Cholinergic synapse	Melanoma	Basal cell carcinoma
Amphetamine addiction	Pathways in cancer	Drug metabolism
Ovarian steroidogenesis	PI3K-Akt signaling pathway	Insulin secretion
Prostate cancer	MAPK signaling pathway	Staphylococcus aureus infection
Regulation of lipolysis in adipocytes	Human cytomegalovirus infection	Aldosterone synthesis and secretion
Cortisol synthesis and secretion	Human papillomavirus infection	Regulation of lipolysis in adipocytes
Retinol metabolism	Coronavirus disease	Glycerolipid metabolism
Adherens junction	Human immunodeficiency virus 1 infection	Long-term potentiation
	Hepatitis B	Renin secretion
	Rap1 signaling pathway	Glioma

Metabolic pathways enriched with upregulated (first column) and downregulated (columns 2 and 3) DEGs in all three TNBC cell lines based on combined treatment. The regulation of these metabolic pathways is associated with the inhibition of cell proliferation in the three cell line models

### Molecular modelling studies

**Fig. 5:**
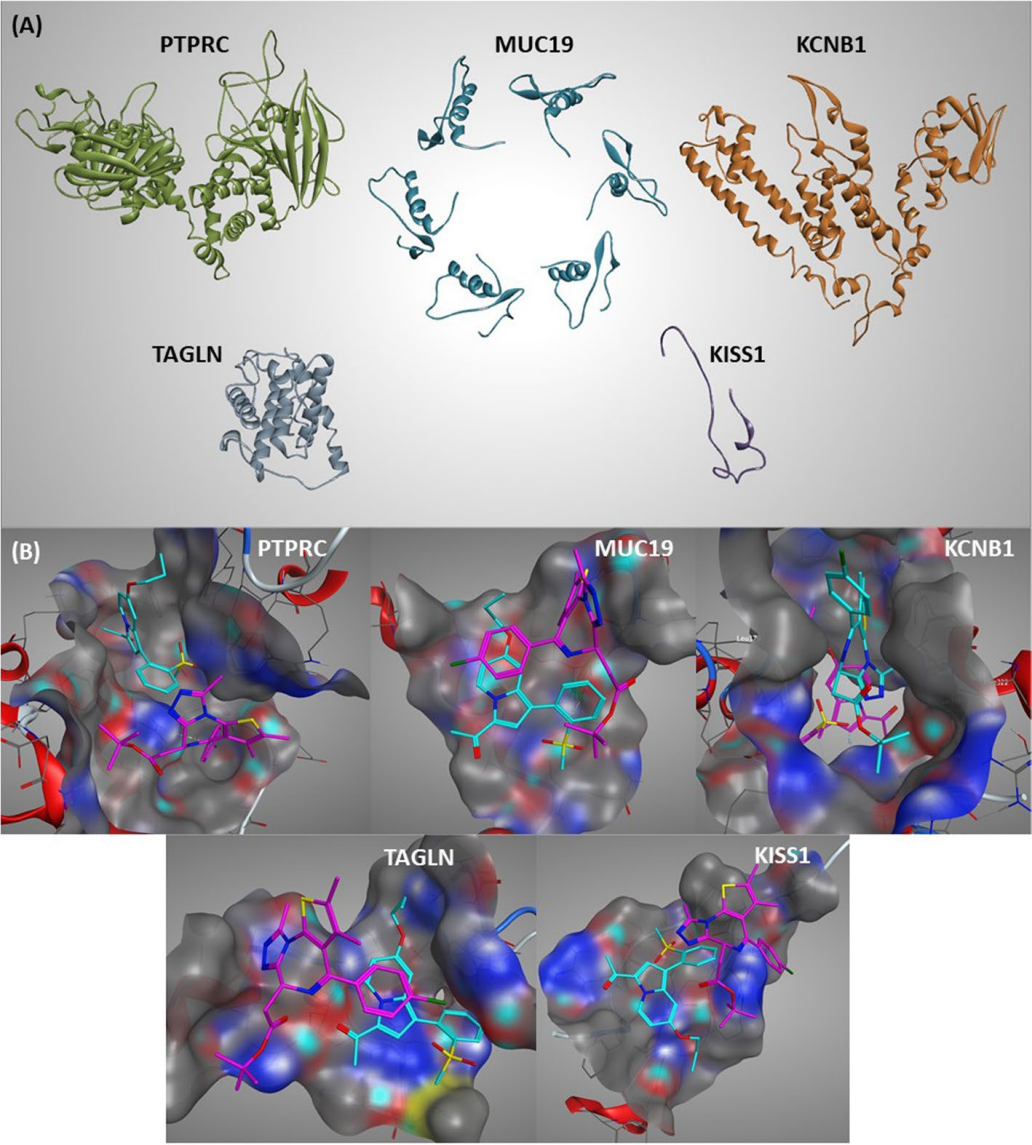
**A.** Homology modelling of PTPRC, MUC19, KCNB1, TAGLN and KISS1 protein. The optimized conformations of five downregulated protein structures represented as cartoon models. The reactive binding domains were constructed for MUC19 and KISS1 proteins due to the lack of template availability. **B.** Molecular docking of JQ1 and GSK2801 against PTPRC, MUC19, KCNB1, TAGLN and KISS1 proteins. Binding mode orientation of JQ1 (Pink) and GSK2801 (Cyan) with downregulated proteins in TNBC. The ligands are shown in the binding site cavities of target proteins

We next sought to explore the impact of JQ1 and GSK2801 on downregulated DEGs at the protein functional level. Our molecular modelling studies supported the inhibitory effect of JQ1 and GSK2801 drugs against TNBC though specific targets. Protein models of PTPRC, MUC19, KCNB1, KISS1 and TAGLN were constructed with validated stereochemical quality explaining their suitability for docking studies (Fig. 5-A). Ramachandran plots are provided in the supplementary material S1 File-Table S6. The optimized conformations of the ligands showed the possibility of hydrogen bonding due to possession of H-bond acceptor atoms. JQ1 and GSK2801 have a hydrophobic surface area of 709.62 Å^2^ and 613.66 Å^2^, respectively, which helps form strong hydrophobic interactions favored by ring structures. Both structures have electronic density clouds on their surface that favor electrostatic interactions with the receptor molecules (Fig. S4).

The molecular docking of JQ1 and GSK2801 provided information regarding the efficiency of their binding and inhibitory action on these target proteins. Both ligands showed the best docking scores, which explains the strong binding affinity with receptor proteins (Table 3). The highest negative score indicates the highest affinity of the ligand. The highest affinity of JQ1 and GSK2801 was found with KISS1, with docking scores of -2.60 and -3.01 kcal/mol, respectively. All the docking scores are between -0.95 to -3.01, which substantiates the stable binding of ligands. The ligands were observed to fit within the binding pockets of the receptor proteins making hydrogen bonds and non-bonded interactions. The ring structures of both ligands favored formation of hydrophobic interactions, which makes the complexes more stable (Fig. 5-B).

**Table 3:** Molecular docking of JQ1 and GSK2801 against downregulated proteins

Protein	Glide Score	
	**JQ1**	**GSK2801**
PTPRC	-1.76	-2.32
MUC19	-1.04	-1.60
KCNB1	-0.95	-2.08
TAGLN	-1.60	-1.69
KISS1	-2.60	-3.01

Glide docking scores of docking complexes of JQ1 and GSK2801 with downregulated proteins in TNBC. These docking scores predict the binding efficiency of drugs.

### Cytotoxicity assays

**Fig. 6:**
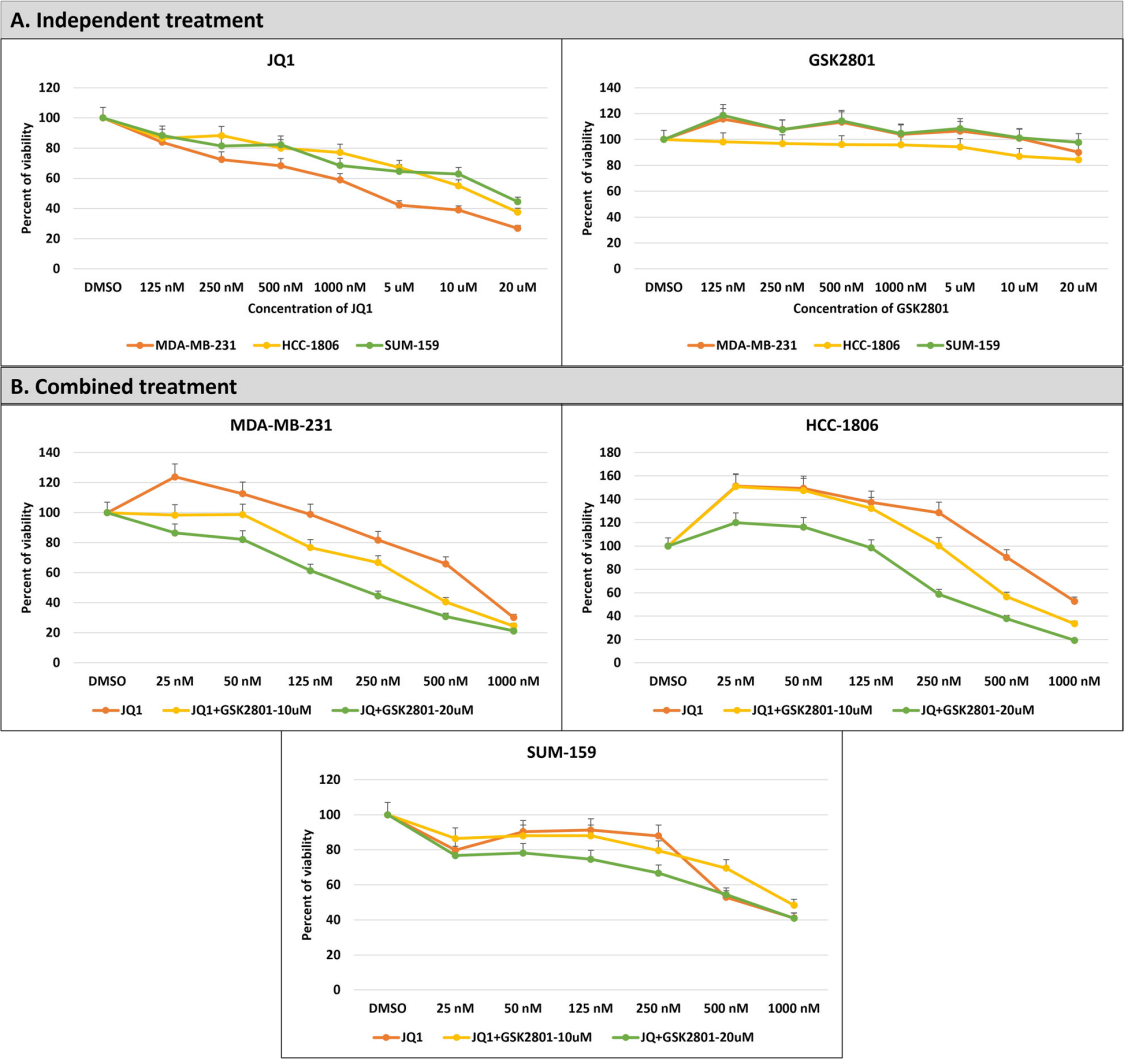
**A.** Cytotoxicity assays of JQ1 and GSK2801 against three TNBC cell lines. **A.** Viability curves explaining the cytotoxic effect of JQ1 and GSK2801 when treated alone on MDA-MB-231, HCC-1806 and SUM-159 TNBC cell lines. **B.** Viability curves explaining the cytotoxic effect of combined treatments of JQ1 and GSK2801 on MDA-MB-231, HCC-1806 and SUM-159 cell lines demonstrating the synergistic effect. Values are given as mean of three independent experiments ± SD. **B.** Quantification of the cell density. Bar plots showing the cell densities measured after the treatment. There is a progressive decrease in the cell density with increasing drug concertation. The cell densities are lower in the combined treatment when compared to single agent treatment

**Fig. 7:**
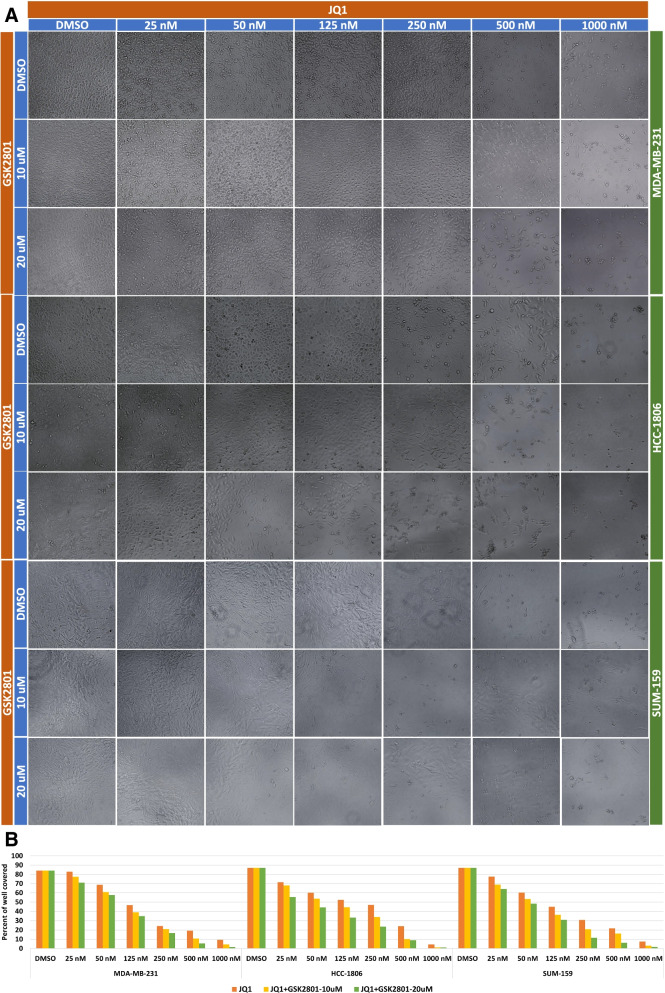
Microscopic images demonstrating the decrease in cell density. With increase in the concentration of drugs (left to right) there is a progressive decrease in density of the cells which indicated a steady death of cancer cells. The cell density is lesser in the combined treatments when compared to the single agent treatment

The independent and additive/synergistic effects of JQ1 and GSK2801 on three different cell lines were determined by MTT assay after 72 h of treatment using varying doses of JQ1 and GSK2801 as explained in the methods section. Dose–response curves of independent and combined treatment were performed, and the viability values were plotted. Administering JQ1 alone showed efficient anti-proliferative activity in all three TNBC cell lines, while GSK2801 alone was ineffective at inducing cytotoxicity at the doses utilized. JQ1 started showing an effect at a concentration of 125 nM and GSK2801 showed a mild effect at a concentration of 20 µM on MDA-MB-231 and HCC-1806 cell lines, but no effect on SUM-159 cells (Fig. 6-A). To examine the potential synergistic effect of JQ1 + GSK2801 combined treatment, the assays were repeated with JQ1 doses of 25 nM, 50 nM, 125 nM, 250 nM, 500 nM and 1000 nM combined with 10 µM or 20 µM of GSK2801. Administering the combined treatment at the previously mentioned doses greatly enhanced the cytotoxic effect within each of the three cell lines. When combined with 10 µM of GSK2801, JQ1 enhanced the cytotoxic effect far more than treatments of JQ1 alone. Using 20 µM of GSK2801 was even more effective at inducing cytotoxicity as compared to 10 µM of GSK2801 when administered in combination with JQ1 (Fig. 6-B). After treatment, microscopic images were captured by EVOS-fl digital inverted microscope under 10X objective and quantified as percent of well of covered using ImageJ software [53]. Variation in cell densities were observed in the treated samples. The cell densities were observed to decrease as the concentration of the drugs increased. Further, the cell densities were reduced to lesser extent in the combined treatment than single agent treatments, which indicates an increase in cancer cell death (Fig. 7). These data show the cytotoxic effects of JQ1 are enhanced by GSK2801, suggesting a potential synergy between these two agents. This synergistic action was further investigated by deriving the CI values for the combined treatment doses (Table 4). The CI values of < 1, = 1, and > 1 indicate synergism, additive effect, and antagonism of drugs, respectively. As shown in Table 4, we found that all the combined doses of JQ1 and GSK2801 showed strong synergistic effect on MDA-MB-231 cells with CI values less than 1. The JQ1/GSK2801 doses at 25 nM/10 µM, 25 nM/20 µM, 50 nM/20 µM showed antagonistic effect on HCC-1806 with CI values > 1, and there appeared to be an additive effect at a dose of 50 nM/10 µM with a CI value of 1. In case of SUM-159 cell lines, most of the lower doses of JQ1 with GSK2801 showed antagonistic effects, whereas the higher doses showed synergistic effect. From the CI indices, it can be observed that JQ1 at lower doses acts antagonistically with GSK2801; however as the concentration of JQ1 increases, so too does its synergistic effect with GSK2801. From the CI index analyses, it appears that JQ1 concentration from 250 nM onwards with 10 and 20 µM of GSK2801 exhibits the strongest synergistic effects, irrespective of the cancer cell line.

**Table 4:** Combination index calculation using Chou and Talalay method in three TNBC cell lines

JQ1 (nM)	GSK2801 (µM)	Combination Index		
		**MDA-MB-231**	**HCC-1806**	**SUM-159**
25	10	0.2	1.2	2.9
50	10	0.2	1.0	3.6
125	10	0.0	0.7	3.6
250	10	0.0	0.4	1.3
500	10	0.0	0.2	0.4
1000	10	0.0	0.1	0.0
25	20	0.2	1.1	1.7
50	20	0.1	1.1	2.1
125	20	0.0	0.7	1.4
250	20	0.0	0.3	0.5
500	20	0.0	0.2	0.1
1000	20	0.0	0.1	0.0

Combination index values calculated for the combined does of JQ1 and GSK2801 on three TNBC cell lines (CI values < 1-synergistic; = 1-additive; > 1-antoagonistic)

### Gene expression analysis

Since anti-cancer activity is generally achieved by the downregulation of cancer-associated genes [54, 55], downregulation of selected genes was assessed by performing RT-qPCR studies. Our analyses of the RNASeq data collected by Bevill et al*.*, (2019) revelated that MDA-MB-231 cells showed downregulation of *TAGLN* and *KISS1* genes (treated with JQ1-100 nM, GSK2801-10 µM, JQ1/GSK2801-100 nM/10 µM) and SUM159 cells showed downregulation of *PTPRC*, *MUC19* and *KCNB1* genes (treated with JQ1-300 nM, GSK2801-10 µM, JQ1/GSK2801-300 nM/10 µM). These results were consistent in our RT-qPCR investigations.

**Fig. 8:**
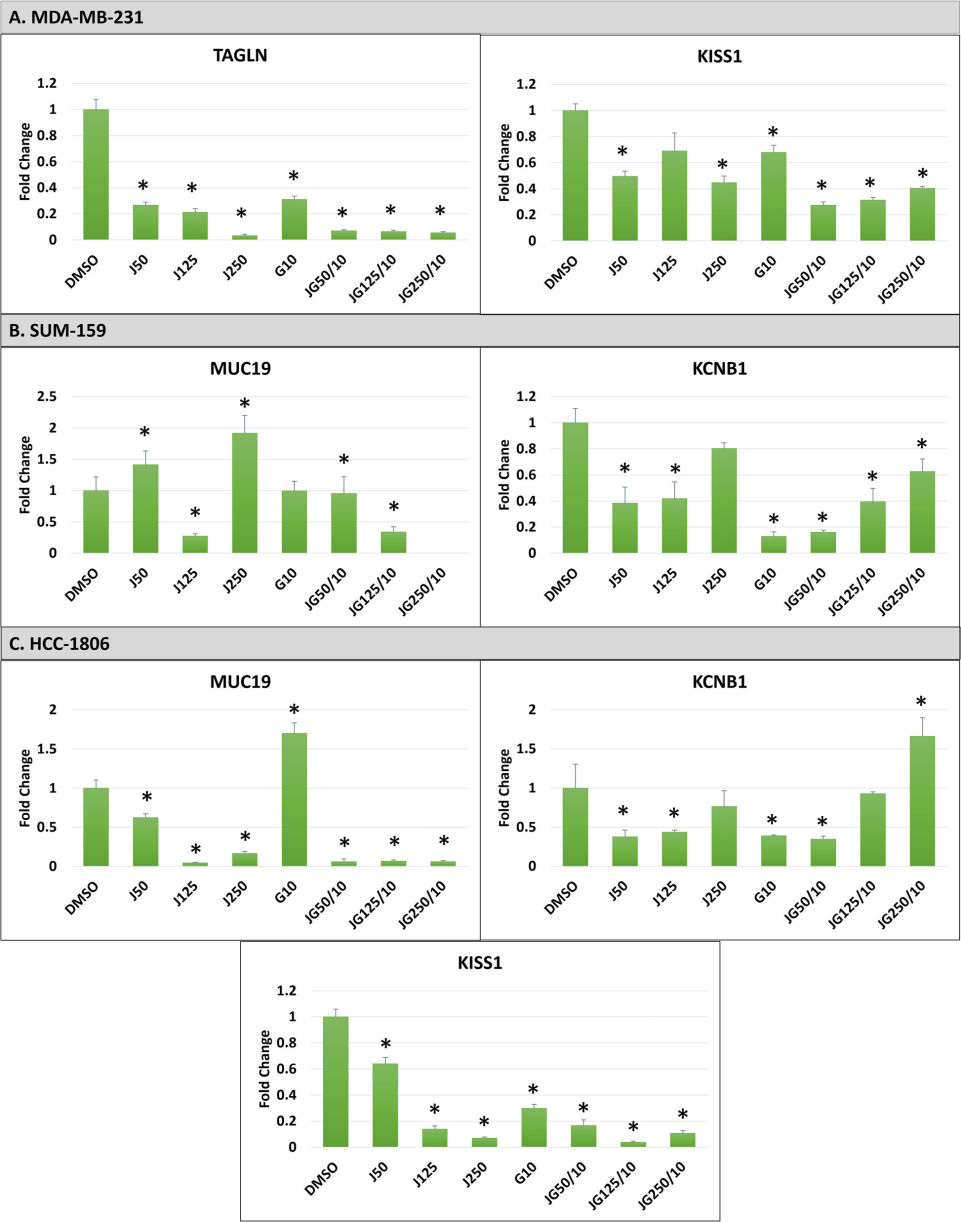
Validation of gene expression in three TNBC cell lines. **A.** Effect of JQ1 and GSK2801 on TAGLN and KISS1 genes in the MDA-MB-231 cells showing the downregulation in the expression both in the single and combined treatments. **B.** Effect of JQ1 and GSK2801 on MUC19 and KCNB1 genes in SUM-159 cells showing the downregulation in the expression. MUC19 was found to be upregulated with a higher concentration of JQ1 (250 nM). **C.** Effect of JQ1 and GSK2801 on MUC19, KCNB1 and KISS1 genes in HCC-1806 cells lines. MUC19 was upregulated by GSK2801 (10 µM). The increase in the JQ1 concentration in the combined treatments increased the expression of KCNB1. KISS1 was observed to be downregulated in all the single and combined treatments. Values are given as mean of three independent experiments ± SD. Statistical significance were defined at *p < 0.05 compared to DMSO control. DMSO: control, J: JQ1, G: GSK2801. JQ1 doses are in nM and GSK2801 in µM

MDA-MB-231: We observed downregulation of *TAGLN* and *KISS1* genes in these cells across all the doses of the single and combined treatments we examined (Fig. 8-A).

SUM-159: We observed the downregulation of *MUC19* and *KCNB1* genes, however we found no expression of PTPRC gene in these cells. *MUC19* was upregulated at the lower (50 nM) and higher doses (250 nM) of JQ1 and downregulated at 125 nM. There was a negligible change in the expression of *MUC19* following GSK2801 treatment. Higher doses of combined JQ1/GSK2801 at 125 nM/10 µM showed downregulation and the expression is undetermined with 250 nM/10 µM of JQ1/GSK2801. *KCNB1* was observed to be downregulated across all the treatment conditions, where lower doses of combined treatment 50 nM/10 µM of JQ1/GSK2801 demonstrated the most significant downregulation of this gene (Fig. 8-B).

HCC-1806: We observed no expression of *PTPRC* and *TAGLN* genes in these cells. *MUC19* was downregulated by JQ1 and combined treatments but was observed to be upregulated when GSK2801 was given alone. KCNB1 was downregulated by JQ1 and GSK2801 and lower doses of the combined treatment. As the JQ1 concentration increased in the combined treatment, so did the expression of *KCNB1*. The results are promising for *KISS1*, where all of the treatments resulted in its downregulation; The combined treatment effect was most prominent with 125 nM/10 µM of JQ1/GSK2801 (Fig. 8-C). These observations suggest that lower doses of JQ1 with 10 µM GSK2801 is the best synergistic dose to treat the HCC-1806 cell lines among the doses we tested. Collectively, these results indicate BD treatments altered gene expression consistent with our RNASeq analysis.

## Discussion

BET and BAZ2A/B domain inhibitors are currently being evaluated at different stages of clinical trials for treating a variety of different cancer types (https://clinicaltrials.gov/; Identifiers: NCT05111561, NCT05053971, NCT05301972, NCT04910152, NCT04471974). Despite modest toxicities, the administration of different BET and BAZ2A/B inhibitors in patients suggest that their co-administration may be more effective than their individual administration [56]. Previous reports on the synergistic combination of BET and BAZ2A/B inhibitors in TNBC preclinical models suggest that they might affect transcriptional regulation via epigenetic mechanisms[57]. This observation has led to the development of targeted inhibitors such as lapatinib and trametinib, which are more effective in tumor inhibition both in vitro and in vivo [58, 59]. Trametinib and Selumetinib are FDA-approved kinase inhibitors. There are also several other kinase inhibitors in various phases of clinical trials, administered alone and in diverse combinations, for targeted therapeutics and chemotherapies (https://clinicaltrials.gov/). The synergistic effects of BET and BAZ2A/B domain inhibitors highlighted here and elsewhere [32] underscore the need for trials of these agents given in combination, similar to what is presently happening with kinase inhibitors.

Among the BD containing proteins, BET and BAZ2A/B domains are the most promising and novel targets for the synergistic action of diverse inhibitors to induce cytotoxicity, as assessed in multiple TNBC cell line models [56, 60–62]. The synergistic action observed with JQ1 and GSK2801 proteins makes a unique combination treatment for TNBC models. Their action may be regulated by transcriptional mechanisms associated with BD containing proteins [33, 63–65]. Our results support a synergistic mechanism of JQ1 and GSK2801 and showed more effective anti-proliferative activity in vitro when these two treatments were given in combination. The probable molecular mechanism for the observed synergy was investigated initially through a computational analysis wherein we identified upregulated and downregulated genes and their associated metabolic pathways.

Several important metabolic pathways associated with cancer were enriched with both upregulated and downregulated genes. Several of these are discussed to illustrate the effectiveness of JQ1 and GSK2801 on the regulation of metabolic pathways resulting in inhibition in TNBC cell lines. Major cancer related pathways enriched with upregulated genes include ABC transporters [66], Rap1 signaling [67], necroptosis [68], ferroptosis [69], PPAR signaling [70] and TGF-beta signaling. Rap1 acts as a switch in the cellular signaling transduction process and Rap1 signaling can regulate cell invasion and metastasis in different cancers. Ferroptosis is a distinct type of regulated cell death where its metabolism and expression of specific genes is associated with the cell inhibition in breast cancer. Activation of genes in the PPAR signaling pathways inhibit tumor progression [71]. Activation of TGF-beta signaling in breast cancer is involved in the suppression of tumor growth [72]. The upregulated pathways in the MDA-MB-231 cell lines were predominantly enriched by the genes such as RRAS, SHC2, TRADD, CAMK2D, GNG7, MAP2K6, PLD1, ITGB3, CCND1, CDKN1A, GNAI1, ITPR1 and PRKACB. These genes are most importantly associated with cancer associated signaling pathways such as cAMP signaling, kinase signaling, NF-kappaB apoptotic signaling, calcium signaling and p38 MAP kinase mediated signal transduction. The upregulated pathways in HCC-1806 were observed to be majorly enriched with the genes such as FOS, GNG7, SHC2, CDKN1A, ITPR1, PLCG2, ITPR2, PRKACB, PRKCB and PIK3R3. These gene are associated with the several important pathways such as growth signaling, B cell activation, apoptosis induction, endothelial cell proliferation, Inositol triphosphate receptor-mediated signaling, transmembrane receptor protein tyrosine kinase signaling and G protein-coupled receptor signaling pathways. SUM-159 cells exhibit enriched expression of CALM1, FOS, CREB3L3, ADCY1 and PIK3R3, associated with growth signaling, regulation of cell proliferation, differentiation, and transformation and calmodulin signaling pathways.

The cancer related pathways enriched with downregulated genes include TNF signaling [73], JAK-STAT signaling [74], IL-17 signaling [75] and NF-kappa B signaling [76] pathways. TNF-α is involved in the epithelial-to-mesenchymal transition and metastasis of breast cancer cells and represents an important target [73]. JAK-STAT signaling represents an important focus as it is a potential therapeutic target to overcome drug resistance [74]. There exists a direct association between IL-17 and breast cancer invasion since IL-17 promotes invasion in some breast cancer cell lines [75]. The NF-kappa B signaling plays a major role by contributing to the aggressiveness of breast cancer and the genes from this pathway represent novel therapeutic targets [76]. The downregulated pathways in the MDA-MB-231 cell lines were predominantly enriched by the genes such as IL1A, PLA2G4A, VEGFA, E2F2, PTGS2, IL12A, NFATC1, NFATC2, EGF, TRAF2, CXCL8, ADCY7, IL1B, IL6, PLCB4 and MAPK13. These genes were observed to be majorly associated with the cancer associated pathways such as MAP kinase, cytokine mediated inflammation, proinflammatory signaling cascade, TNF-induced apoptosis, T cell receptor stimulation and mitogenic pathways. The downregulated pathways of HCC-186 were majorly enriched by the genes such as IKBKE, PDGFB, IL12A, IL1A, IRAK1, ITGB2, MYC, CALML4, ADCY7, TLR4, CALML5 and IL1B. These genes are associated with the several important pathways such as cytokine mediated inflammation, calmodulin, Toll-like receptor, cell cycle progression, apoptosis and cellular transformation, IL1-induced upregulation of NF-kappa B and oncanonical I-kappa-B pathways. Finally, the downregulated pathways of SUM-159 cell lines were predominantly enriched with the genes such as ITGB2, PDGFB, PDGFRA, PDGFRB, TLR2, RAC3, TGFB2, ADCY7, PLCB1 and MAPK3. These genes are associated with the metabolic activities such as MAP kinase signaling, TGF-beta signaling and Toll-like receptor signaling pathways. Collectively, these signaling, and metabolic pathways all contribute to cell proliferation and aggressive properties in TNBC. It is noticed that no metabolic pathways were enriched with upregulated and downregulated genes from GSK2801 single agent treatment in MDA-MB-231 and HCC-1806 cell lines whereas only one pathway i.e., PPAR signaling was enriched with the upregulated genes of GSK2801. Such findings most likely reflect heterogeneity between tumor cell lines.

*PTPRC*, which belongs to protein tyrosine phosphatase (PTP) family involves in the regulation of variety of cellular processes including: cell growth, differentiation, mitosis, and oncogenic transformation making it useful as a prognostic or predictive biomarkers and/or direct target [77]. PTPRC acts as essential regulator of T-and -B-cell antigen receptor signaling and associated with lymphocyte-specific immune recruitment thereby plays a major role in patient survival [78, 79]. *MUC19* reportedly modulates proliferation, invasion and metastatic potential of breast cancer [80]. MUC19 expression was notably observed to increase in TNBC and found to be targeted by miR-593 [81]. *KCNB1* is a complex class of voltage-gated ion channels and its overexpression was reported as biomarker in breast cancer [82]. *TAGLN* is a potentially useful diagnostic marker differentially expressed in TNBC and a potential target for treatment strategies [83]. TAGLN was reported to be frequently downregulated by DNA hypermethylation and its promotor methylation profiles also serves as diagnostic markers with a possible clinical impact in the TNBC [84, 85]. *KISS1* is of tremendous utility in controlling metastasis in a therapeutic context [86]. KISS1 is known as a downstream target of the canonical TGFβ/Smad2 pathway and has been identified as a putative human metastasis suppressor gene in in TNBC [87, 88]. JQ1 and GSK2801 were able to modulate their expression triggering their downregulation and inducing the cytotoxicity in TNBC cell lines models. The results from the MTT assay and RT-qPCR studies support the inhibitory activity of JQ1 and GSK2801. We further sought to predict their inhibitory action at the protein structural level through molecular modelling methods to theoretically support their inhibitory action by means of deriving their binding energies and intermolecular interactions with the target proteins. The comprehensive binding analysis of JQ1 and GSK2801 suggest their use as combinatorial drugs because of their similar binding interactions at the defined binding sites of the target proteins.

So far, the most tractable inhibitor for BET is JQ1 [31] and for BAZ2A/B is GSK2801 [32]. Inhibition of such domains noticeably contributed to the cytotoxicity of the JQ1 + GSK2801 combinatorial treatments. Use of this combination enabled us to induce anti-proliferative activity more efficiently in the three TNBC cell line models along with the plausible identification of the target genes and the mechanisms.

TNBC has not previously been associated with significant mutations or copy number alterations in the BET or BAZ2A/B domains. This paves the way to develop inhibitors against BD as therapeutics and represents an ideal choice to target TNBC. The distinct functional roles of BET and BAZ2A/B domains in TNBC suggests inhibition of their BD may result in selective tumor toxicities. Our findings explain how co-inhibition of BET and BAZ2A/B BD represent an effective approach to regulate transcription of *TAGLN*, *KISS1*, *PTPRC*, *MUC19* and *KCNB1* genes thereby inducing anti-proliferative activity. BD are likely to mediate several important cancer pathways that suppress oncogenic pathologies. It is important to identify the strategies to improve the clinical utilities of BDI following the conclusions of their phase I studies to examine their safety. Overall, the BDI represent promising combinatorial agents and further studies into their mechanisms of action would enable us to identify the most effective combinations to fast-track their use as therapeutic drugs.

In the current study, the expression of five downregulated genes were characterized through molecular modelling and RT-qPCR, anticipating that the downregulation is associated with cancer inhibition. Additionally, the alteration of metabolic pathways requires additional investigation. Finally, the effective concentrations of JQ1 and GSK2801 require in vivo evaluation.

Overall, the current study advances our understanding of the potential targets of JQ1/GSK2801 and underscores the combinatorial use of BDI against TNBC. Effective doses of JQ1/GSK2801 were determined for the three different TNBC cell lines, which provides motivation for future in vivo studies. The five downregulated genes characterized in the current study may serve as biomarkers of BDI sensitivity in TNBC. Our studies present coinhibition of JQ1 and GSK2801 in combination as an effective strategy to induce cytotoxicity and arrest the progression of TNBC. While our studies support the synergistic role of JQ1 and GSK2801, the functional analysis of the candidate genes in BET/BEZ inhibition along with additional in vivo evaluations is the important next step of the current study. These investigations will help to advance the present study to determine the possible molecular mechanism involved in cell inhibition and also to conclude the synergistic activity of the drugs beyond the validation of candidate gene expressions.

## Conclusions

The combined cytotoxic effect of JQ1 and GSK2801 on MDA-MB-231, HCC-1806 and SUM-159 cells has been documented through MTT assays. Our findings provide experimental support for the hypothesis that combined JQ1-GSK2801 shows synergistic growth inhibitory activity on TNBC cell line models. Further, we observed downregulated expression of cancer associated genes such as *PTPRC, MUC19, KCNB1, TAGLN* and *KISS1*, validated through RT-qPCR studies, with additional support from molecular modelling studies. These results from the current study suggest the combination of JQ1 and GSK2801 may be effective in the treatment of TNBC. Further in vivo investigations through mouse xenograft models would help to standardize the accurate synergistic doses to move towards clinical trials. The results from the current study may warrant further investigation as a potential treatment for TNBC.

## Supplementary Information

**Additional file 1: S1 file. **This file contains thefigures and tables generated during the data analysis and is available at https://doi.org/10.7910/DVN/BEW3OR. **Fig.S1.** Analysis of gene counts. Boxplot (A) before and (B) after normalization explaining the distribution of genecounts; (C) Heat map of samples (D) Heat map of gene counts among the samples. **Fig. S2.** Multidimensional analysis of samples. Multidimensional scalingplots before (left) and after (right) normalization explaining the distributionof control and treated samples among three different breast cancer cell lines. **Fig.S3.** Number of upregulated anddownregulated metabolic pathways. The number of upregulated anddownregulated pathways in the threedifferent treatment conditions (JQ1, GSK2801 and JQ1 +GSK2801) across threedifferent TNBC cell lines. The unique and shared number of pathways amongdifferent treatment conditions are represented. **Fig. S4.** Optimized conformation of JQ1 and GSK2801. The structures are represented in stickmodel with the hydrogen bond donor (blue) and acceptor (red) surface areas. Theelectron density clouds are represented in green dots. **Table S1**. Table. RNASeq data sets. RNASeq expression data retrieved from GEOdatabase. Three different TNBC cell lines were treated with JQ1 and GSK2801alone and in combination for 72 hours. DMSO was used as a vehicle and served asan internal control. **Table S2.**The genes and primers. Primers usedfor the evaluation of gene expression in the breast cancer cell lines underdifferent treatment conditions. **Table S3-S5.** Smear plots and volcano plots. DEGs observed inMDA-MB-231, HCC-1806 and SUM-159 cell lines among different treatmentconditions. Green dots represent downregulated and red dots upregulated genes. **Table S6.** Ramachandran plots. The stereochemicalvalidations were done by generating the Ramachandran plots for the homologymodels of five downregulated proteins.

**Additional file 2: S2 file. **The Gene count matrix. The gene counts generated from the RSEM analysis showing a list of 44,567genes corresponds all the three different TNBC (MDA-MB-231, HCC-1806 andSUM-159) cell lines treated with three treatment conditions (JQ1, GSK2801 andJQ1 +GSK2801). Available at https://doi.org/10.7910/DVN/GMSXLN.

**Additionalfile 3: S3 file. **Differentially expressed genes (DEGs). DEGs observed from three TNBC cell lines. The DEGSare separated as upregulated and downregulated genes based on the log2 foldchange and p-values. Available at https://doi.org/10.7910/DVN/0SI5AB.

**Additionalfile 4: S4 file. **Grouping of DEGs.The upregulated and downregulated genes from three different TNBC cell lines aregrouped based on the three treatment conditions to find out the common andunique genes among the treatments. Available at https://doi.org/10.7910/DVN/BJFDD8.

**Additionalfile 5: S5 file. **Gene enrichment analysis. Metabolic pathways enriched with upregulated anddownregulated genes in three different TNBC cell lines and three differenttreatment conditions. Available at https://doi.org/10.7910/DVN/KWVOJV.

## Data Availability

The RNASeq datasets analyzed in the current study are from Bevil et al*.,* 2019 and are available in the NCBI-GEO repository https://www.ncbi.nlm.nih.gov/geo/query/acc.cgi?acc=GSE116907 The research data generated during the analysis in the current study are available from the Harvard Datavserse repository. The description of each file and their repository links are provided as follows.

## Abbreviations

TNBC: Triple-Negative Breast Cancer
ER: Estrogen Receptor
PR: Progesterone Receptor
HER2: Human Epidermal Growth Factor Receptor 2
BDI: Bromodomain Inhibitors
BD: Bromodomains
BET: Bromodomain-Extra-Rerminal domain
CREB: Cyclic-AMP Response Element Binding protein
CBP: CREB Binding Protein
BAZ2A: Bromo Adjacent to Zinc Finger 2A
BAZ2B: Bromo Adjacent to Zinc Finger 2B
DEG: Differentially Expressed Genes
GEO: Gene Expression Omnibus
SRA: Short Read Archive
TMM: Trimmed Mean of M-values
logFC: Log2 Fold Change
logCPM: Log Counts Per Million
KEGG: Kyoto Encyclopedia of Genes and Genomes
NCBI: National Center for Biotechnology Information
OPLS: Optimized Potentials for Liquid Simulations
FBS: Fetal Bovine Serum
PTP: Protein Tyrosine Phosphatase
MTT: (3-(4,5-Dimethylthiazol-2-yl)-2,5-diphenyltetrazolium bromide
DMSO: Dimethyl Sulfoxide
OD: Optical Density
nm: Nano meters
nM: Nano moles
µM: Micro moles
MDS: Multidimensional Scaling
