# Supplementary material for: Synergistic anti-proliferative activity of JQ1 and GSK2801 in triple-negative breast cancer
Source: BMC Cancer. 2022 Jun 8;22:627. doi: 10.1186/s12885-022-09690-2 (PMC9173973; doi:10.1186/s12885-022-09690-2)
Supplement: Supplementary file 1 — Additional file 1: S1 file. This file contains thefigures and tables generated during the data analysis and is available at https://doi.org/10.7910/DVN/BEW3OR. Fig.S1. Analysis of gene counts. Boxplot (A) before and (B) after normalization explaining the distribution of genecounts; (C) Heat map of samples (D) Heat map of gene counts among the samples. Fig. S2. Multidimensional analysis of samples. Multidimensional scalingplots before (left) and after (right) normalization explaining the distributionof control and treated samples among three different breast cancer cell lines. Fig.S3. Number of upregulated anddownregulated metabolic pathways. The number of upregulated anddownregulated pathways in the threedifferent treatment conditions (JQ1, GSK2801 and JQ1 +GSK2801) across threedifferent TNBC cell lines. The unique and shared number of pathways amongdifferent treatment conditions are represented. Fig. S4. Optimized conformation of JQ1 and GSK2801. The structures are represented in stickmodel with the hydrogen bond donor (blue) and acceptor (red) surface areas. Theelectron density clouds are represented in green dots. Table S1. Table. RNASeq data sets. RNASeq expression data retrieved from GEOdatabase. Three different TNBC cell lines were treated with JQ1 and GSK2801alone and in combination for 72 hours. DMSO was used as a vehicle and served asan internal control. Table S2.The genes and primers. Primers usedfor the evaluation of gene expression in the breast cancer cell lines underdifferent treatment conditions. Table S3-S5. Smear plots and volcano plots. DEGs observed inMDA-MB-231, HCC-1806 and SUM-159 cell lines among different treatmentconditions. Green dots represent downregulated and red dots upregulated genes. Table S6. Ramachandran plots. The stereochemicalvalidations were done by generating the Ramachandran plots for the homologymodels of five downregulated proteins. [file 12885_2022_9690_MOESM1_ESM.docx]

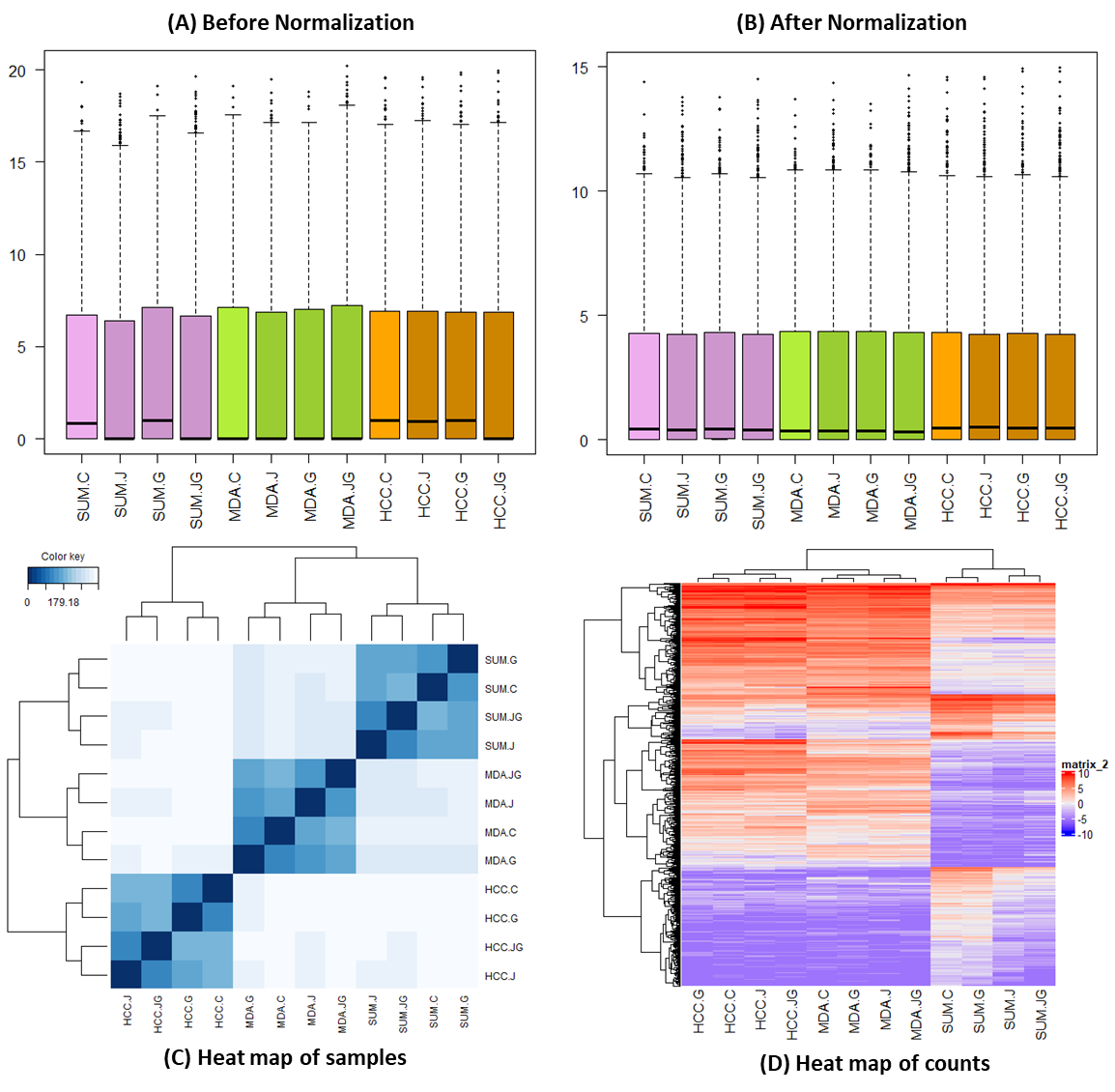


**Figure S1**. Box plot **(A)** before and **(B)** after normalization explaining the distribution of gene counts; **(C)** Heat map of samples **(D)** Heat map of gene counts among the samples.

Cell lines: MDA: MDA-MB-231; HCC: HCC-1806; SUM: SUM-159

Treatments: J: JQ1; G: GSK2801; JG: JQ1 & GSK2801


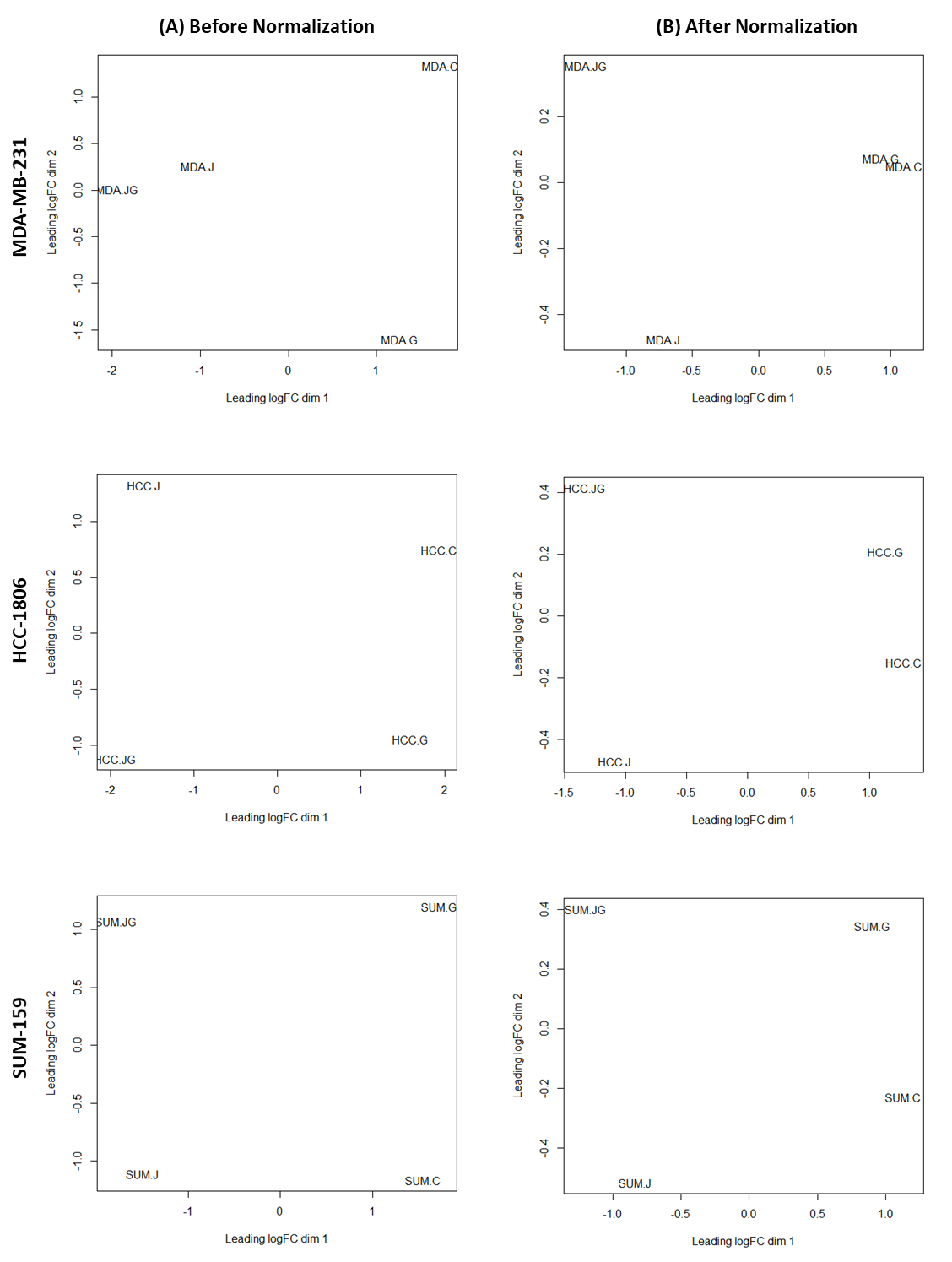


**Figure S2.** Multidimensional scaling plots before (left) and after (right) normalization explaining the distribution of control and treated samples among three different breast cancer cell lines.

Cell lines: MDA: MDA-MB-231; HCC: HCC-1806; SUM: SUM-159

Treatments: J: JQ1; G: GSK2801; JG: JQ1 & GSK2801


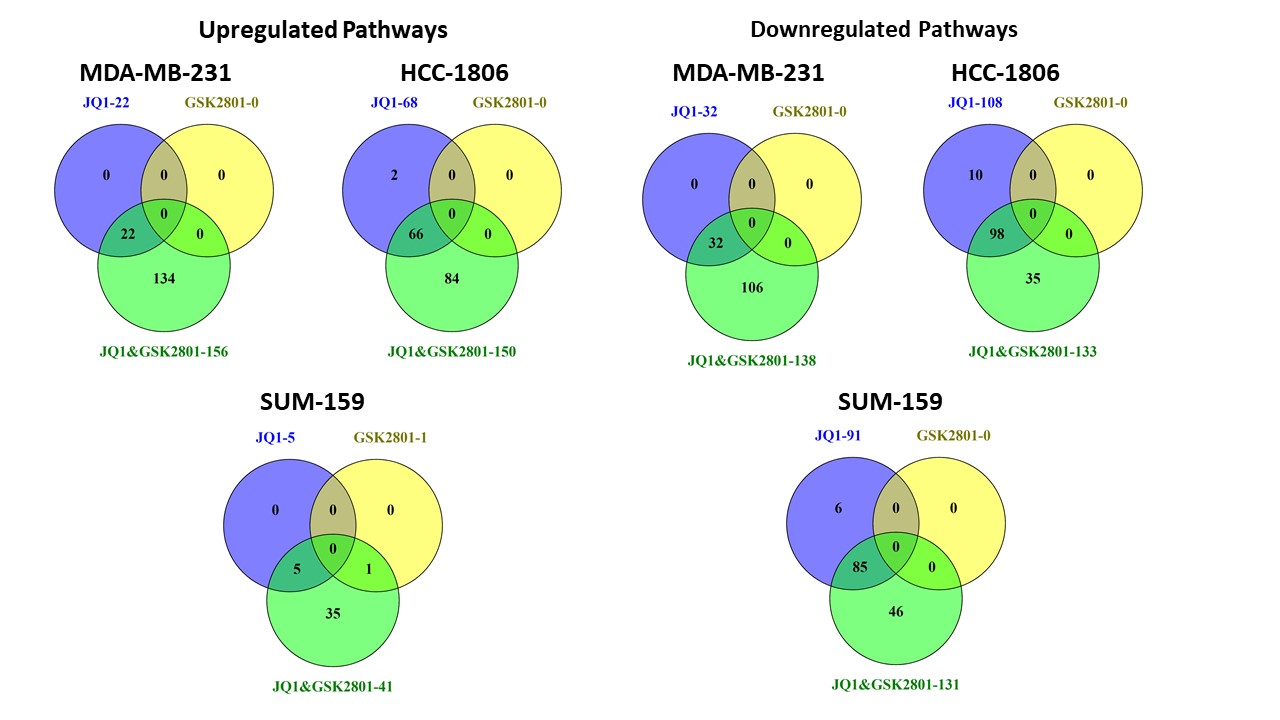


**Figure S3**. **Number of up and downregulated metabolic pathways.** The number of upregulated and downregulated pathways in the three different treatment conditions (JQ1, GSK2801 and JQ1&GSK2801) across three different TNBC cell lines. The unique and shared number of pathways among different treatment conditions are represented.


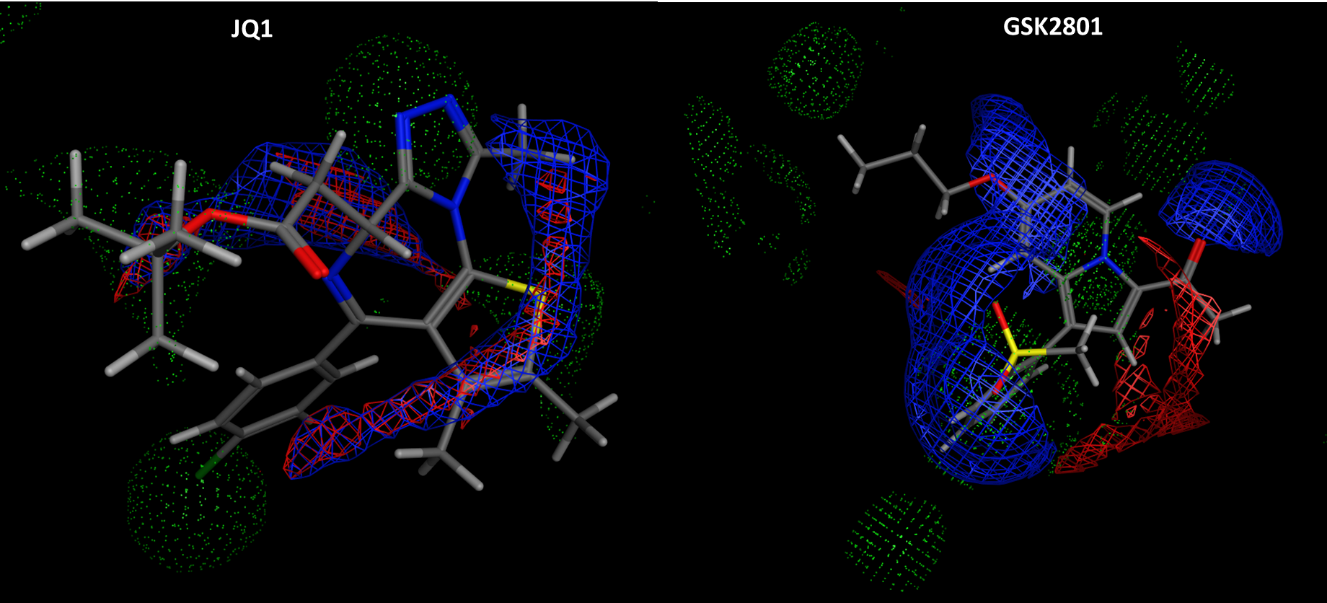


**Figure S4.** **Optimized conformation of JQ1 and GSK2801.** The structures are represented in stick model with the hydrogen bond donor (blue) and acceptor (red) surface areas. The electron density clouds are represented in green dots.

**Table S1. RNASeq data sets.** RNASeq expression data retrieved from GEO database. Three different TNBC cell lines were treated with JQ1 and GSK2801 alone and in combination for 72 hours. DMSO was used as a vehicle and served as an internal control.

| **GEO ID** | **SRA ID** | **Cell lines** | **Treatment** |
| --- | --- | --- | --- |
| GSM3263568 | SRR7508559 | SUM159 | DMSO |
| GSM3263569 | SRX4378999 |  | JQ1-300nM |
| GSM3263570 | SRX4379000 |  | GSK2801-10uM |
| GSM3263571 | SRX4379001 |  | JQ1-300nM & GSK2801-10uM |
| GSM3263572 | SRX4379002 | MDAMB231 | DMSO |
| GSM3263573 | SRX4379003 |  | JQ1-100nM |
| GSM3263574 | SRX4379004 |  | GSK2801-10uM |
| GSM3263575 | SRX4379005 |  | JQ1-100nM & GSK2801-10uM |
| GSM3263576 | SRX4379006 | HCC1806 | DMSO |
| GSM3263577 | SRX4379007 |  | JQ1-500nM |
| GSM3263578 | SRX4379008 |  | GSK2801-10uM |
| GSM3263579 | SRX4379009 |  | JQ1-500nM & GSK2801-10uM |

**Table S2. The genes and primers.** Primers used for the evaluation of gene expression in the breast cancer cell lines under different treatment conditions.

| **Gene** | **Primers 5’🡪3’** | **Product size** |
| --- | --- | --- |
| **PTPRC** | F: TTCATGCAGCTAGCAAGTGG  R: AGTCAGCCGTGTCCCTAAGA | 116 |
| **MUC19** | F: CACCTTCAGAGACTGCCACA  R: TCACATGCACAGGCTTCTTC | 78 |
| **KCNB1** | F: AGGCGAGGAGTTCGATAACA  R: ATGGTGGAGAGGACGATGAA | 135 |
| **TAGLN** | F: GGCCAAGGCTCTACTGTCTG  R: CCTCCAGCTCCTCGTCATAC | 142 |
| **KISS1** | F: CCCACCCTCTGGACATTCAC  R: CCTAGAAGTGCCTTGAGGCTTG | 105 |
| **18S rRNA** | F: GTAACCCGTTGAACCCCATT  R: CCATCCAATCGGTAGTAGCG | 151 |

**Table S3.** Smear plots and volcano plots representing the DEGs in **MDA-MB-231** cell lines among different treatment conditions. Green dots-downregulated, red dots-upregulated genes.

| JQ1 | 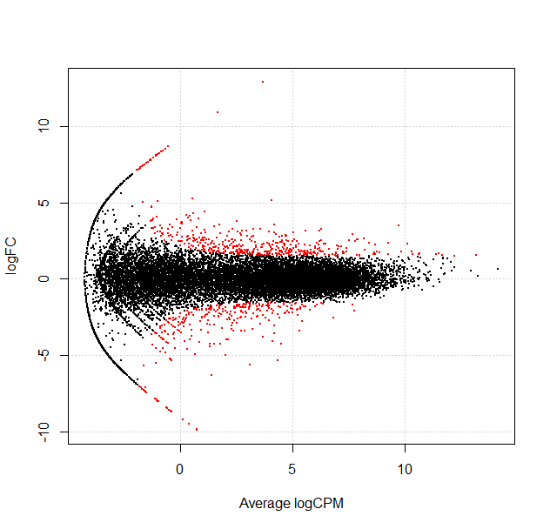 | 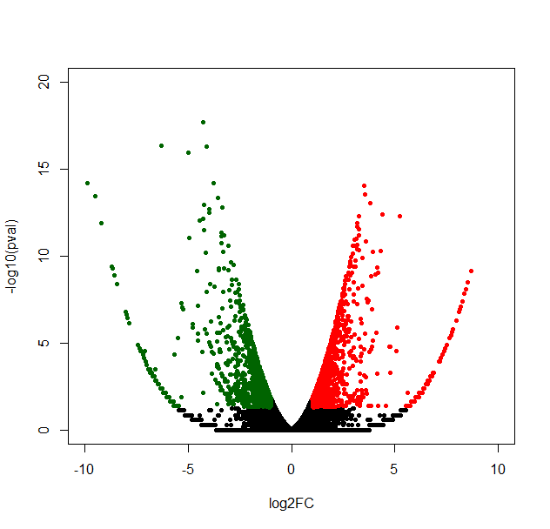 |
| --- | --- | --- |
| GSK2801 | 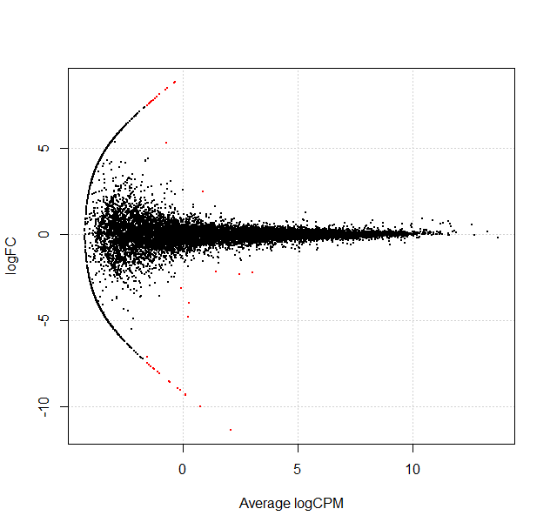 | 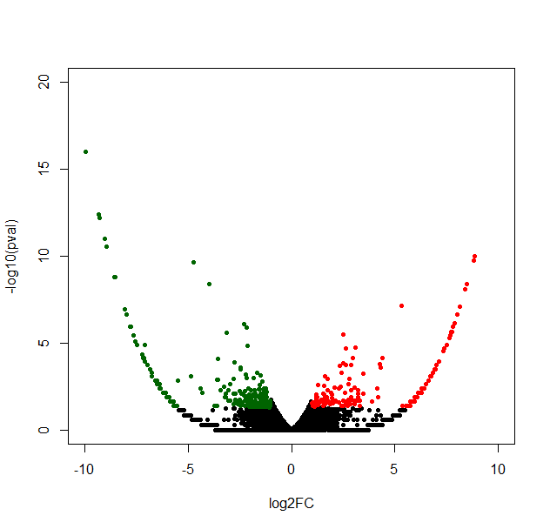 |
| JQ1& GSK2801 | 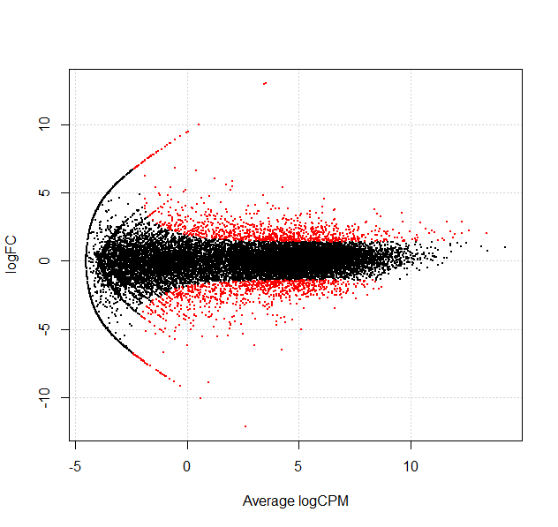 | 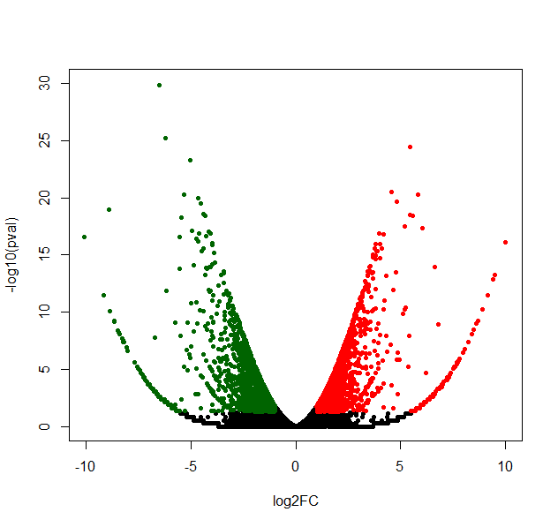 |

**Table S4.** Smear plots and volcano plots representing the DEGs in **HCC-1806** cell lines among different treatment conditions. Green dots-downregulated, red dots-upregulated genes.

| JQ1 | 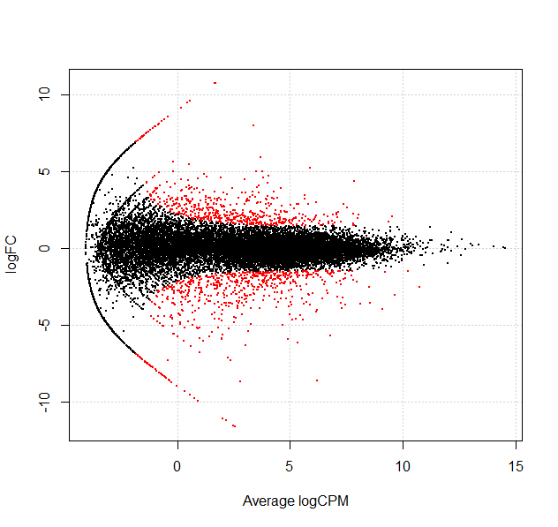 | 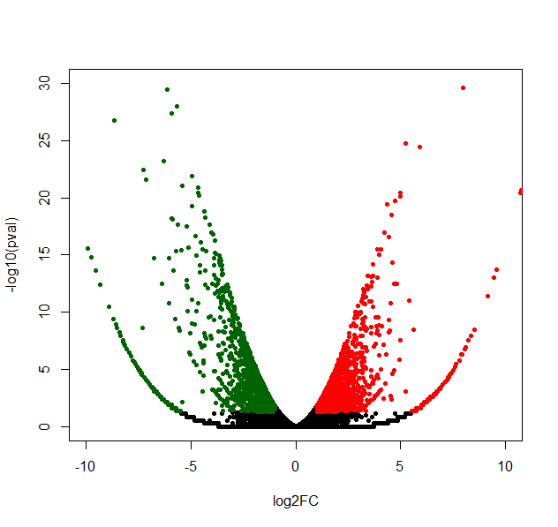 |
| --- | --- | --- |
| GSK2801 | 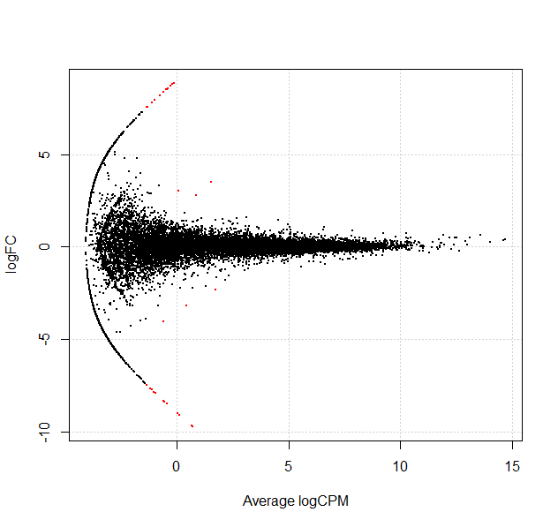 | 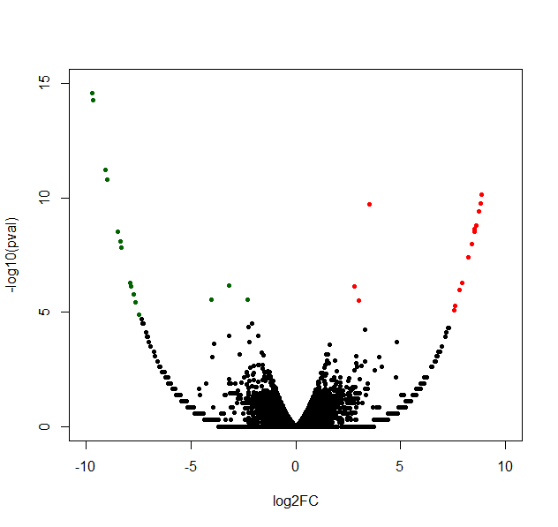 |
| JQ1& GSK2801 | 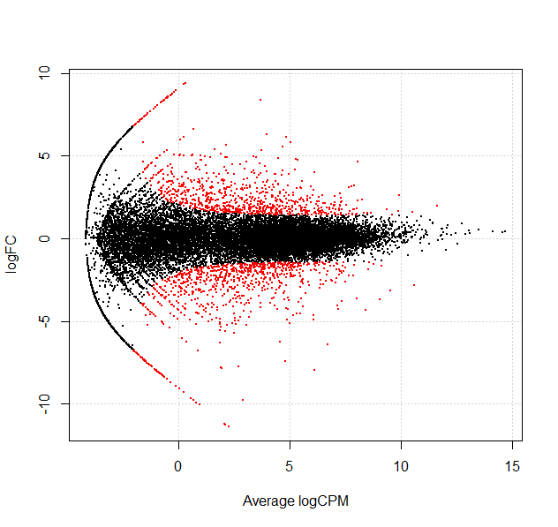 | 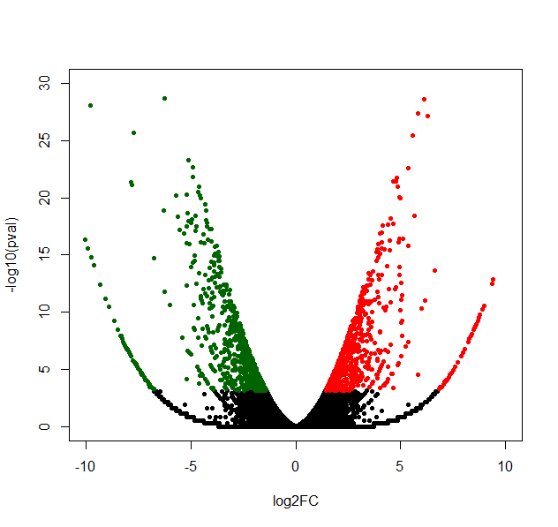 |

**Table S5.** Smear plots and volcano plots representing the DEGs in **SUM-159** cell lines among different treatment conditions. Green dots-downregulated, red dots-upregulated genes.

| JQ1 | 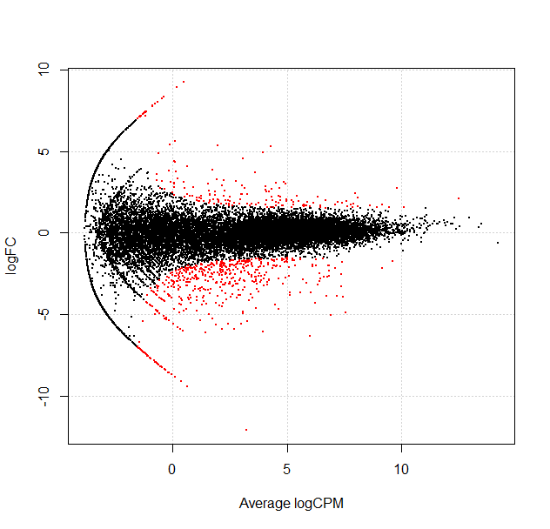 | 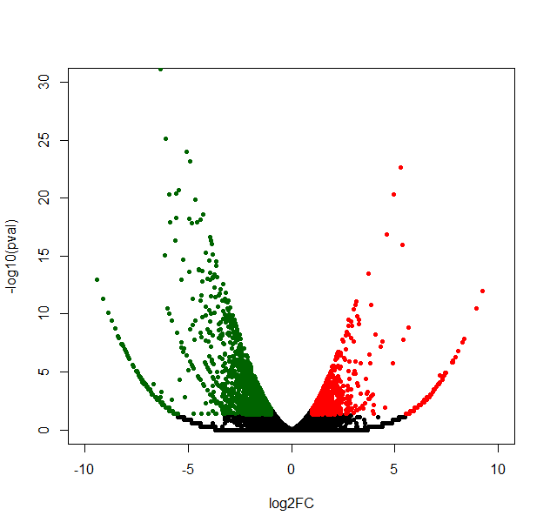 |
| --- | --- | --- |
| GSK2801 | 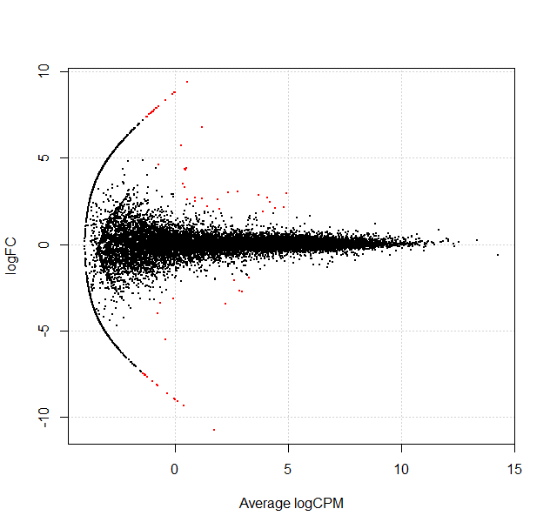 | 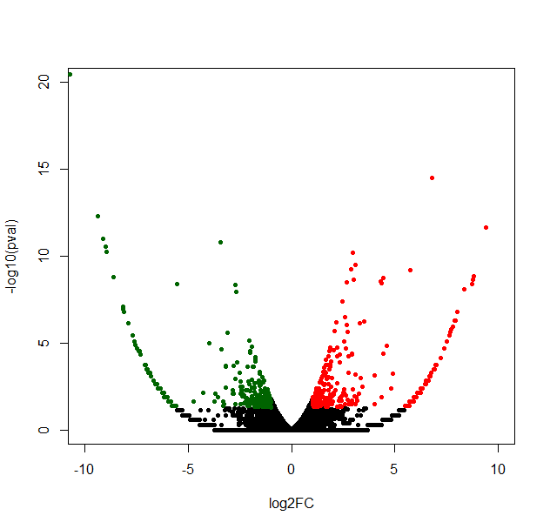 |
| JQ1& GSK2801 | 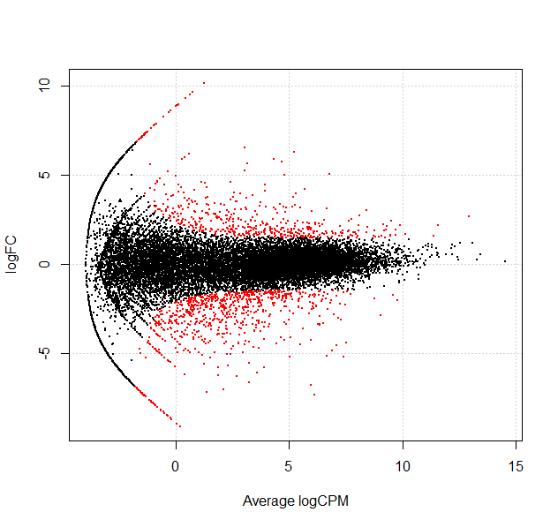 | 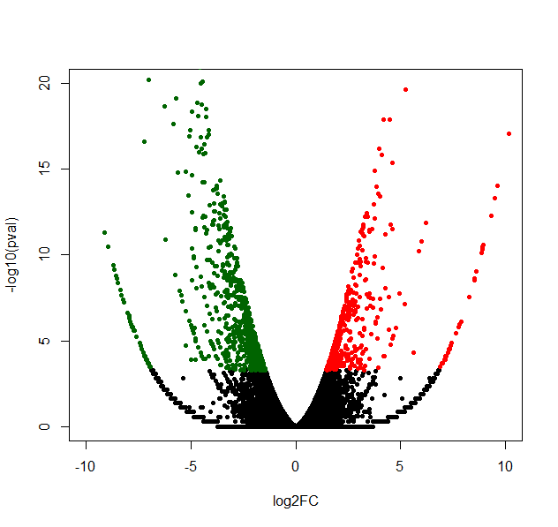 |

**Table S6**. Ramachandran plots explaining the valid stereochemical quality of five down regulated proteins.

| **Protein name** | **Ramachandran plot** |
| --- | --- |
| **PTPRC** | 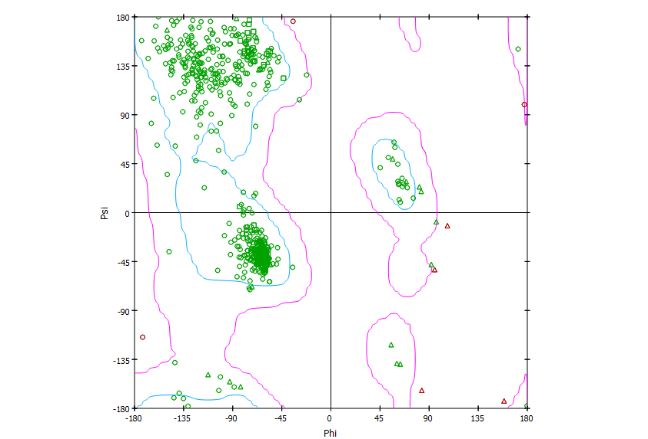 |
| **MUC19** | 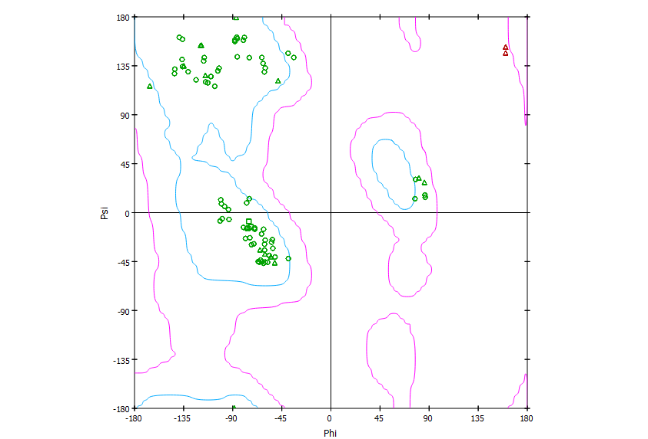 |
| **KCNB1** | 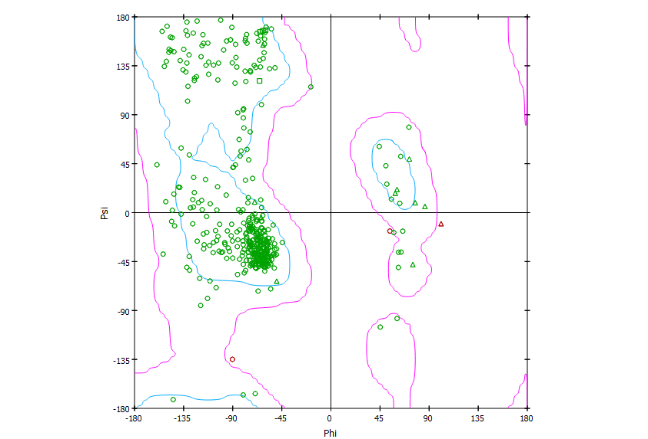 |
| **TAGLN** | 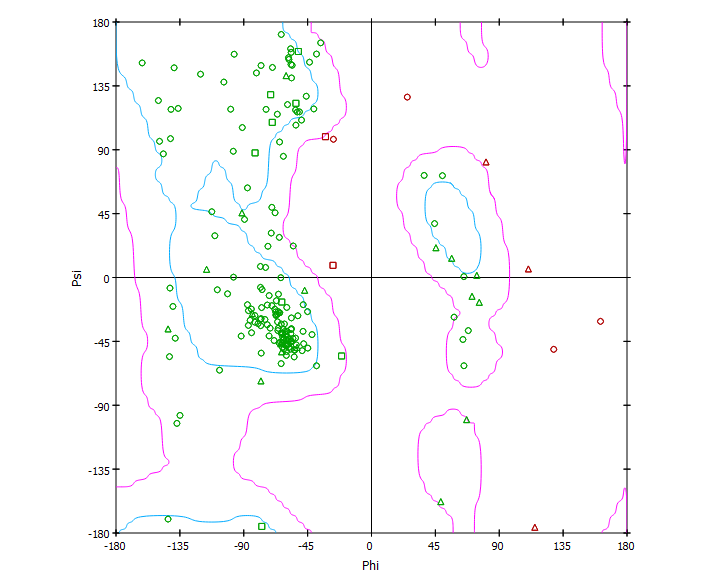 |
| **KISS1** | 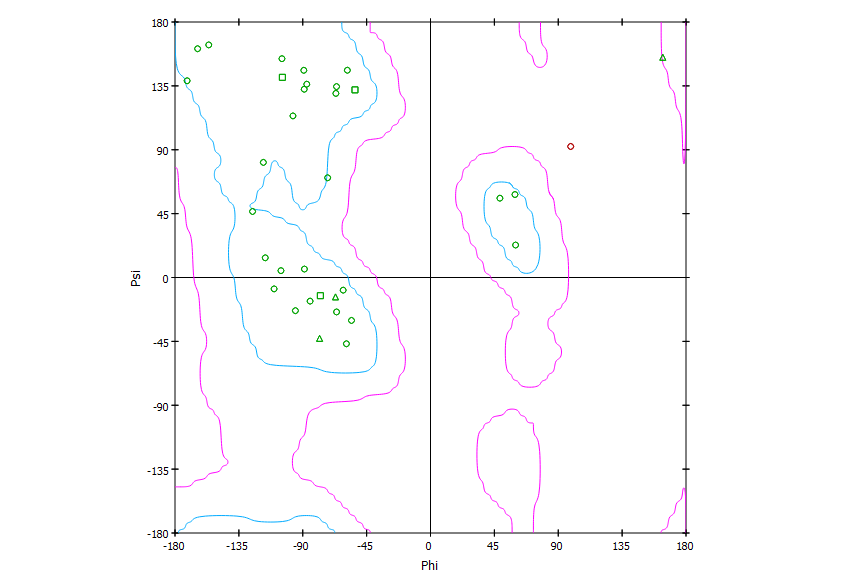 |
