# Supplementary material for: Synergistic anti-proliferative activity of JQ1 and GSK2801 in triple-negative breast cancer
Source: BMC Cancer. 2022 Jun 8;22:627. doi: 10.1186/s12885-022-09690-2 (PMC9173973; doi:10.1186/s12885-022-09690-2)

**Supporting information S4 File**. Differentially expressed genes (DEGs) clustered into upregulated and downregulated genes in three different treatment conditions where the commonly/uniquely regulated elements are identified. Further, it is clearly observed that, the combined treatment (Green shaded area) is showing a greater number of DEGs than other two treatment conditions. The common upregulated and downregulated elements are represented in dotted circles and the list is provided in the dotted boxes. As these elements were observed to be upregulated and downregulated in all the three treatment conditions (JQ1, GSK2801 and JQ1&GSK2801), their importance in the anti-proliferation activity was further investigated through *in vitro* studies.

**MDA-MB-231**:


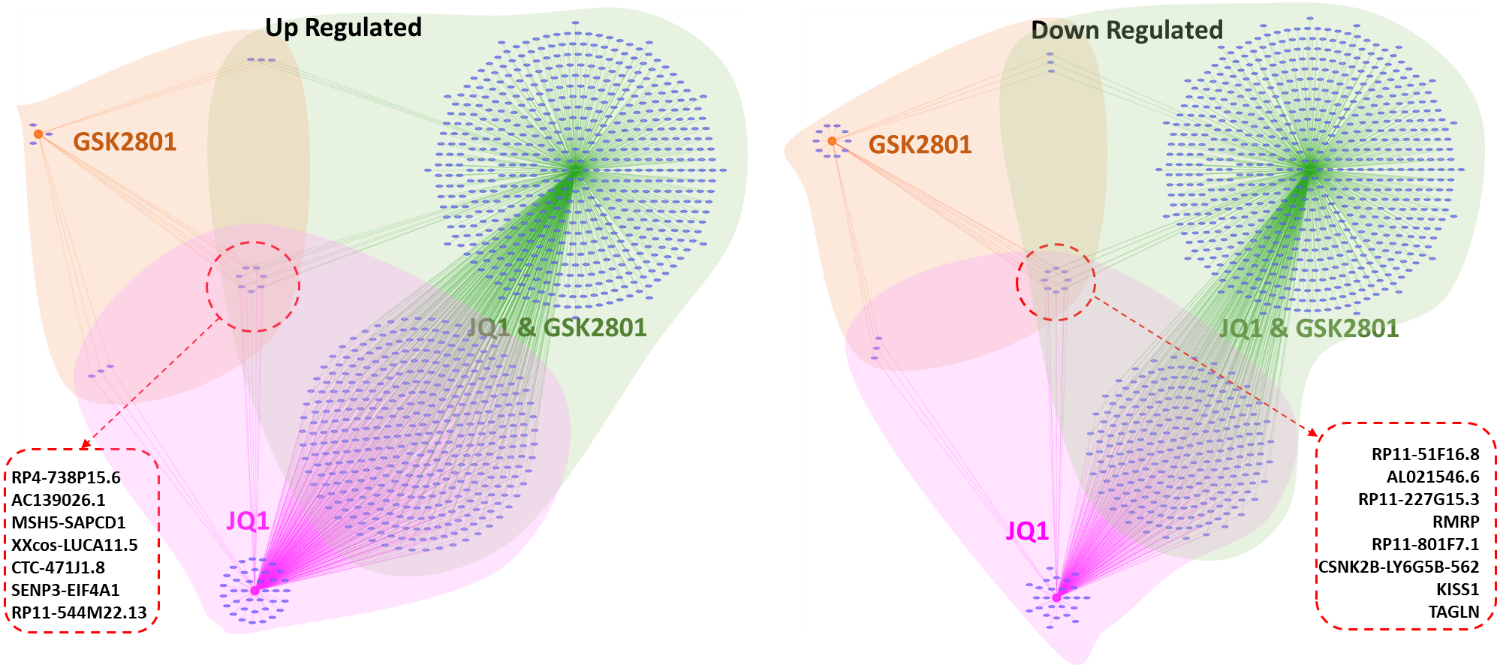


**HCC-1806**:


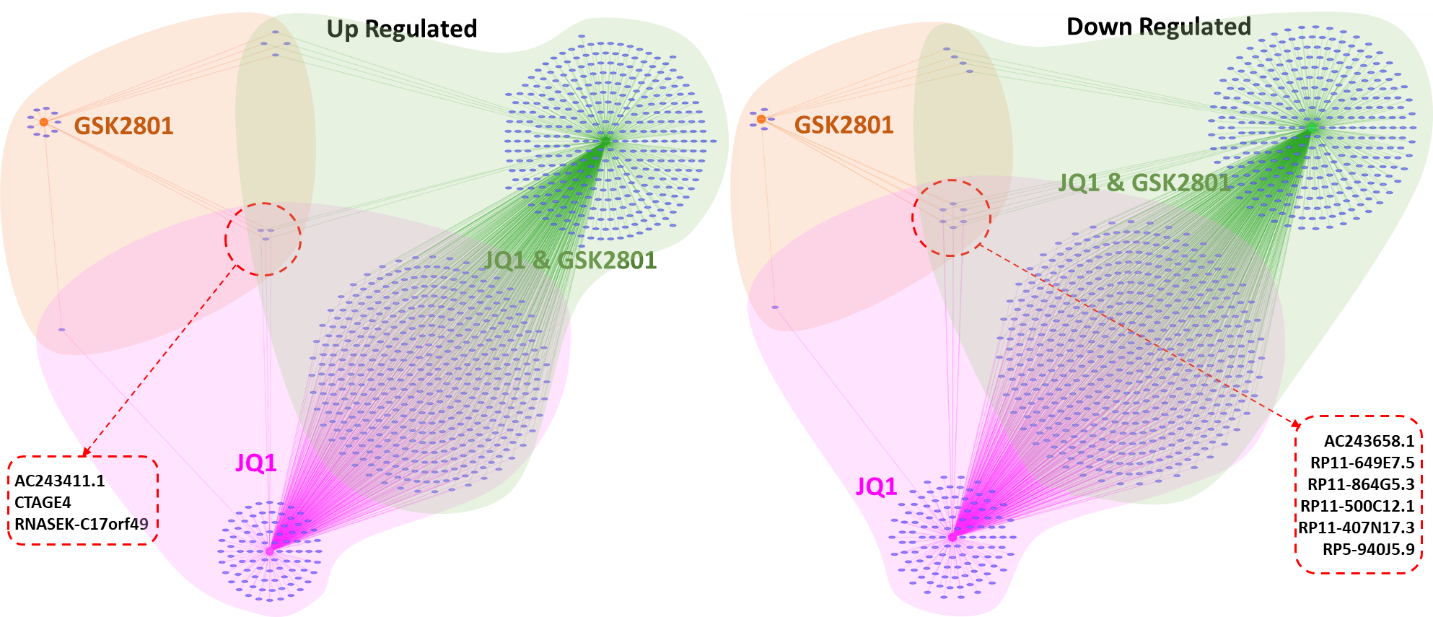


**SUM-159**:


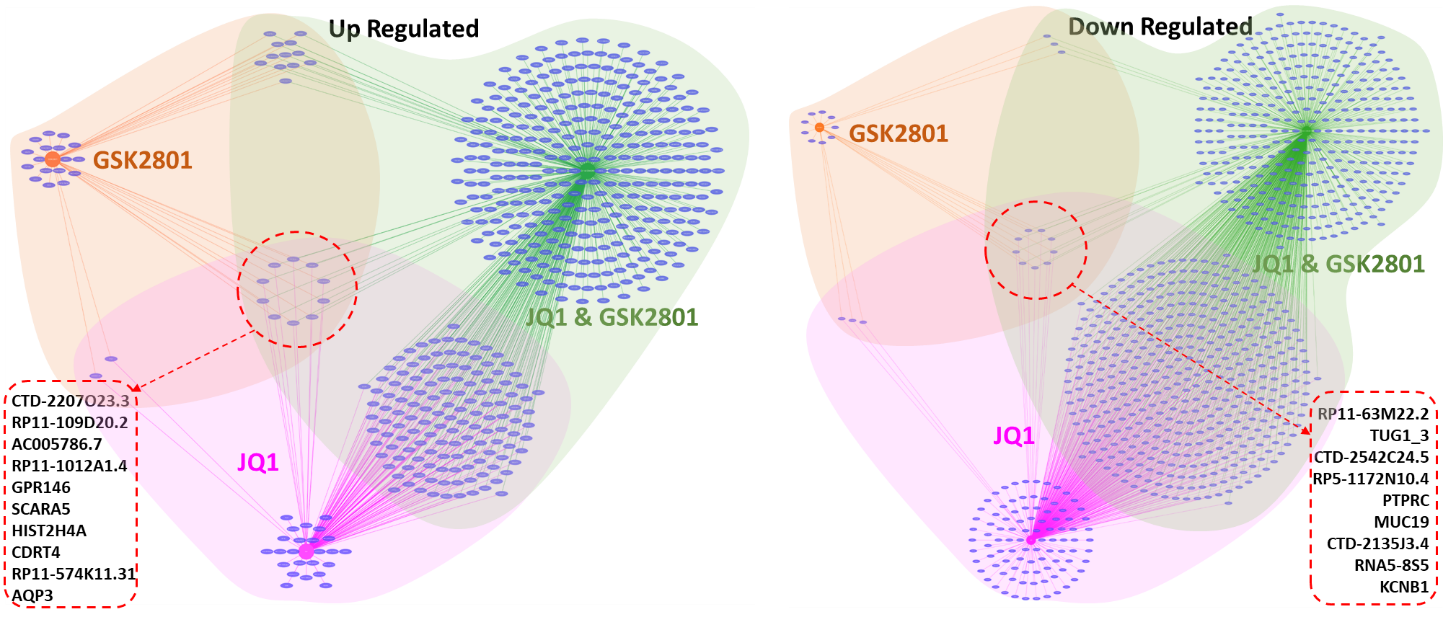

Supplement: Supplementary file 4 — Additionalfile 4: S4 file. Grouping of DEGs.The upregulated and downregulated genes from three different TNBC cell lines aregrouped based on the three treatment conditions to find out the common andunique genes among the treatments. Available at https://doi.org/10.7910/DVN/BJFDD8. [file 12885_2022_9690_MOESM4_ESM.docx]
